# Parallel mRNA and MicroRNA Profiling of HEV71-Infected Human Neuroblastoma Cells Reveal the Up-Regulation of miR-1246 in Association with *DLG3* Repression

**DOI:** 10.1371/journal.pone.0095272

**Published:** 2014-04-16

**Authors:** Li-Juan Xu, Tao Jiang, Wei Zhao, Jian-Feng Han, Juan Liu, Yong-Qiang Deng, Shun-Ya Zhu, Yue-Xiang Li, Qing-Gong Nian, Yu Zhang, Xiao-Yan Wu, E-De Qin, Cheng-Feng Qin

**Affiliations:** 1 Department of Virology, State Key Laboratory of Pathogen and Biosecurity, Beijing Institute of Microbiology and Epidemiology, Beijing, China; 2 PLA 404 Hospital, Weihai, Shandong, China; 3 School of Public Health and Tropical Medicine, Southern Medical University, Guangzhou, Guangdong, China; 4 Graduate School, Anhui Medical University, Hefei, Anhui, China; University of Hong Kong, Hong Kong

## Abstract

Human enterovirus 71 (HEV71) has emerged as the leading cause of viral encephalitis in children in most Asian countries. The roles of host miRNAs in the neurological pathogenesis of HEV71 infection remain unknown. In the present study, comprehensive miRNA expression profiling in HEV71-infected human neuroblastoma SH-SY5Y cells was performed using the Affymetrix Gene Chip microarray assay and was validated using real-time RT-PCR. Among the 69 differentially expressed miRNAs, miR-1246 was specifically induced by HEV71 infection in human neuroblastoma cells, but inhibition of miR-1246 failed to affect HEV71 replication. Parallel mRNA and microRNA profiling based on the 35 K Human Genome Array identified 182 differentially regulated genes. Target prediction of miR-1246 and network modeling revealed 14 potential target genes involved in cell death and cell signaling. Finally, a combined analysis of the results from mRNA profiling and miR-1246 target predication led to the identification of disc-large homolog 3 (DLG3), which is associated with neurological disorders, for further validation. Sequence alignment and luciferase reporter assay showed that miR-1246 directly bound with the 3′-UTR of DLG3 gene. Down-regulation of miR-1246 induced significant changes in DLG3 expression levels in HEV71-infected SHSY5Y cells. Together, these results suggested that miR-1246 might play a role in neurological pathogenesis of HEV71 by regulating DLG3 gene in infected cells. These findings provide new information on the miRNA and mRNA profiles of HEV71-infected neuroblastoma cells. The biological significance of miR-1246 and DLG3 during the course of HEV71 infection deserves further investigation.

## Introduction

Human enterovirus71 (HEV71) is a single-stranded, positive-sense RNA virus belonging to the genus *Enterovirus*, family *Picornaviridae* [1], [2]. HEV71 has been identified as one of the leading pathogensof hand-foot-and-mouthdisease (HFMD) and is associated with encephalitis, meningitis, and neurological complications, even death, in infants and young children. Since the 1998 outbreak in Taiwan, HEV71 has become a newly emerging, life-threatening pathogen in children, specifically in the Asia-Pacific region [3]. Millions of cases, including hundreds of deaths, are confirmed annually in mainland China. Thus far, no vaccine or effective antiviral therapy is commercially available.

The neuropathogenesis of HEV71 infection remains largely elusive. Previous studies have provided rigorous radiological or histopathological evidence regarding the induction of paralysis via the infection and destruction of the anterior horn motor neurons of the spinal cord [4], [5]. Some cases of HEV71-mediated paralysis may induce acute flaccid paralysis by several mechanisms, leading to the virus-mediated destruction of anterior horn motor neurons [6], [7]. HEV71 radiculomyelitis is most frequently observed in the medulla oblongata, reticular formation, pons, and midbrain structures by magnetic resonance imaging and post-mortem studies [8], [9]. Other studies in cynomolgus macaques demonstrated that the development of paralytic disease following HEV71 infection was associated with inflammatory infiltrates in the spinal cord and medulla oblongata [10], [11]. Importantly, HEV71 viral antigen has been found in neurons following immunocytochemical staining [12], [13].

miRNAs are a class of non-coding, single-stranded RNAs 18–25 nucleotides in length that are found in the genomes of all multicellular organisms and some viruses[14]. Functionally, miRNAs prevent the translation of mRNAs or result in the degradation of mRNAs by binding to complementary sequences in the mRNA[15]; in this way, miRNAs work to regulate the cell cycle, cell differentiation, proliferation, development, apoptosis, oncogenesis, and immunity[16]–[20]. Cellular miRNAs may be used to regulate the replication of viruses and reshape the cellular gene expression environment to regulate the tissue tropism of viruses *in vivo* [21], [22]. Additionally, viral miRNAs may directly regulate the viral and/or host cell gene expression to benefit the viruses [23]–[25]. Thus, the expression of host miRNAs during HEV71 infection is the focus of much interest. Cui *et al.* performed comprehensive miRNA profiling in HEV71-infected Hep2 cells (human laryngeal cancer cell) using deep sequencing technology; they then compared the host serum miRNA levels in patients with HFMD caused by HEV71 or coxsackievirus type A16 (CV-A16), as well as healthy individuals [26], [27]. The microarray assay for miRNA profiling in HEV71-infected RD cells (human muscle cell) identified the involvement of hsa-miR-141 during HEV71 infection [28]. Additionally, miRNA profiling in HEV71-infected Vero cells indicated that hsa-miR-296-5p inhibited HEV71 replication by targeting the viral genome [29], and hsa-miR-23b inhibited HEV71 replication through the down-regulation of the HEV71 VP1 protein[30]. However, the host miRNA response to HEV71 infection in human nerve cells remains unknown.

To date, joint genome-wide profiling of mRNA and miRNAs in HEV71-infected nerve cells is still lacking. Our previous study showed that differentially regulated mRNAs are involved in cell cycle/proliferation, apoptosis, and cytokine/chemokine responses [31]. In an effort to understand host cellular regulation during HEV71 infection, we performed comprehensive miRNA and mRNA microarray profiling in HEV71-infected human neuroblastoma cells. The results demonstrated that miR-1246 specifically responds to HEV71 and other enterovirus infections in SH-SY5Y cells. Additionally, up-regulation of miR-1246 reduced the levels of disc-large homolog 3 (*DLG3*), which was associated with neurological disorders, particularly X-linked mental retardation (XLMR). These findings provided new information on the miRNA and mRNA profile of HEV71-infected nerve cells and highlighted the important roles of miRNAs in the neuropathogenesis of HEV71 infection.

## Materials and Methods

### Viruses and Cells

Human rhabdomyosarcoma cells (RD, ATCC, CCL-136), baby hamster kidney cells (BHK-21, ATCC, CCL-10), Human hepatocellular liver carcinoma cells (HepG2, ATCC, 77400) and African green monkey kidney cells (Vero, ATCC, CCL-81) were grown in Dulbecco’s modified Eagle’s medium (DMEM, Gibco, USA) supplemented with 10% fetal bovine serum (FBS, Gibco), 50 U/mL penicillin, and 0.1 mg/mL streptomycin at 37°C in 5% CO_2_. Human neuroblastoma cells (SH-SY5Y,ATCC, CRL-2266) were cultured in DMEM/F-12 medium (Gibco), supplemented with 10% FBS, 50 U/mL penicillin, and 0.1 mg/mL streptomycin at 37°C in 5% CO_2_.

HEV71 strain AH08/06 [32], poliovirus type 3 (PV-3) strain Sabin, Coxsackievirus type B5 (CV-B5) strain CC10/10 [33], and Japanese encephalitis virus (JEV) strain SA14 [34] were used in the current study. HEV71, PV-3, and CV-B5 were propagated in RD cells, and JEV was propagated in BHK-21 cells. The viral stocks were prepared, and the virus titers were determined using a plaque assay as previously described [34], [35].

### Virus Infection

For virus infections of HEV71, PV-3, CV-B5, and JEV, SH-SY5Y, RD, and Vero cells were seeded in 6-well plates, respectively. After overnight incubation, the infections were carried out at a multiplicity of infection (MOI) of 1. After 1 hour incubation, the cells were washed with PBS, and medium containing 2% FBS was added to the cells. The SH-SY5Y cells were harvested for microarray and quantitative RT-PCR (qRT-PCR) assay at 6 and 12 hours post-infection (hpi).

### MicroRNA Array Analysis

To screen miRNA expression during HEV71 infection, miRNA profiles were analyzed using the Affymetrix GeneChip miRNA Array2.0 (CapitalBio Corporation, Beijing, China) according to the manufacturer’s instruction. Briefly, miRNAs were purified from total RNA extracted from HEV71-infected cells or mock-infected cells and were then labeled using an Enzyme Linked Oligosorbent Assay (ELOSA) QC Assay and hybridized to the miRNA array. The array data were normalized by global normalization using the miRNA QC Tool software. The levels of miRNAs between the HEV71-infected and control samples were calculated based on the fluorescence intensities. Differential expression levels of miRNAs between the two groups of samples were assessed using a one-way ANOVA analysis.

### Validation of Selected miRNAs Using qPCR

The qRT-PCR assay of selected miRNAs, including hsa-miR-1246, hsa-miR-125a, hsa-miR-320b, hsa-miR-494, hsa-miR-1268, and hsa-miR-211, was performed using the miScript Reverse Transcription kit and miScript SYBR Green PCR kit (QIAGEN) according to the manufacturer’s instructions with specific miRNA primers from QIAGEN. The HEV71-infected SH-SY5Y cells together with the mock infection cells were collected and lysed, and miRNAs were isolated using the miRNA isolation kit (QIAGEN) according to the manufacturer’s instructions. The amount of each miRNA relative to the endogenous reference RNA U6 was described using the equation 2^−ΔΔCt^.

### Evaluation of miR-1246 by qRT-PCR Assay

To confirm the specific expression of miR-1246 in HEV71-infected cells, the infected RD, Vero, and SH-SY5Y cells, along with the control cells, were collected. In addition, SH-SY5Y cells infected with HEV71, PV-3, CV-B5, and JEV, along with the control cells, were collected to evaluate the miR-1246 expression in response to other enterovirus infections. The miRNAs were isolated from the samples using a miRNA isolation kit (QIAGEN), and the miR-1246 level was quantified using qRT-PCR according to the QuantiTec SYBR Green PCR Kit (QIAGEN). Data were subjected to statistical analysis to assess the difference between the two groups; a *P* value <0.05 was considered to be statistically significant.

### SH-SY5Y Cells Infected with HEV71 after Transfection with miR-1246 Inhibitor

The inhibitorof miR-1246 (MIN0005898) and its negative control oligonucleotides (1027271) were purchased from QIAGEN. The SH-SY5Y cells were seeded at 6×10^4^ cells/well in 24-well plates shortly before transfection. Cells were transiently transfected using the HiPerFect Transfection Reagent (QIAGEN) according to the manufacturer’s instructions. Each well contained the miR-1246 negative control (50 nM, final concentration) or the miR-1246 inhibitor (100 nM, final concentration). At 12 hour after transfection, the cells were infected with HEV71 at an MOI of 1. At 6 and 12 hpi, the supernatants were collected for virus titer analysis by qRT-PCR assay.

### mRNA Expression Profiling

The mRNA gene expression profiling of SH-SY5Y cells infected with HEV71 was carried out using the 35 K Human Genome Array (Operon), which comprised ∼70 bp oligonucleotide probes for 35035 genes from the human genome Oligodatabase (human_V4.0) (CapitalBio). Firstly, SH-SY5Y cells were transiently transfected with the miR-1246 inhibitor or the negative control using the HiPerFect Transfection Reagent (QIAGEN) according to the manufacturer’s instructions. At 12 hpi, the cells were lysed with TRIzol (Invitrogen) and frozen for mRNA profiling analysis according to the manufacturer’s protocol. All data were submitted to the GEO microarray database according to LuxScan 3.0 standards (CapitalBio). All files were transformed and normalized using Loess normalization techniques. The degree of fold-change (relative fluorescence intensity) was analyzed for all of the differentially regulated genes. The significant genes list was determined for hierarchical clustering.

### Computational Analysis Validating the miR-1246 Targets and mRNAs

Potential targets of miR-1246 were predicted using miRanda and TargetScan6.0. The mRNA target pairs that were up- or down-regulated >1.5-fold or <−1.5-fold (*P*<0.05) were then selected for further analyses using the CapitalBio Molecule Annotation System V3.0 (CMAS3.0) (http://bioinfo.capitalbio.com/mas3/). Predicted interactions represent both direct and indirect associations that are derived from various sources, including GenBank, EMBL, SwissProt, Gene Ontology, KEGG, and BioCarta. The cut-off values for the inclusion in these analyses included a differential gene expression, with p-value <0.05, and a fold change >1.5 or <−1.5 (based on SAM) [36].

Selected co-occurrence genes were further analyzed by qRT-PCR; GAPDH was used as an internal control. Real-time RT-PCR primers were designed using the Beacon Designer software (Table S5). The qRT-PCR reaction was performed according to the manufacturer’s protocol. Each assay was performed in triplicate.

### Inhibition of miR-1246 in HEV71-infected Cells

SH-SY5Y cells were infected with HEV71 followed by transfection with varying doses of miR-1246 inhibitor. The cells were then harvested, and qRT-PCR was performed to determine the relative level of *DLG3* transcription.

### Construction of miR-1246 Expression Plasmid

Two complementary oligonucleotides were designed and synthesized based on the cDNA sequence of the *Homo sapiens* miR-1246 precursor including restriction enzyme sites as well as protecting bases. The two annealed complementary oligonucleotides were then ligated into the *pSilencer* 2.1-U6 hygro expression vector. The recombinant plasmid, pS-miR-1246, was confirmed by restriction enzyme digestion and DNA sequencing.

### Luciferase Reporter Assay

To create a luciferase reporter construct, the 3′-UTR segments of DLG3 that contained the putative binding sites for miR-1246 were generated by PCR using the following primers: 5′-tcattctagatcatcatgtgactgtgcc-3′, and 5′-ccatggccggcctacgttgcaccgttcaga-3′. The purified PCR products were cloned into the pGL3-C (Promega, Madison, WI) vector downstream of the luciferase gene to generate the pGL3-DLG3 construct. Site-directed mutagenesis of the predicted miR-1246 target sites in the 3′-UTR of DLG3 was generated with the following primers: 5′-ctctgtacctaattgcacctgtgctagcgcttgggaaa-3′ and 5′-aggtgcaattaggtacagagccattgtttt-3′. The construct was confirmed by sequencing and named pGL3-DLG3-mut. For reporter assay, HepG2 cells cultured in 24-well plates were co-transfected with pS-miR-1246 and pGL3-DLG3 using Lipofectamine 2000 (Life Technologies). The plasmids pGL3-C and pGL3-DLG3-mut were set as control. The pRL-CMV plasmid (Promega) expressing *Renilla luciferase* was co-transfected to normalize the transfection efficiency. Then, cells were harvested 48 h after transfection, and Firefly and *Renilla luciferase* activity levels were measured using the Dual-luciferase Reporter Assay (Promega, Madison, WI) according to the manufacturer’s instruction. Each assay was performed in triplicate.

### Statistical Analysis

The data were analyzed using Microsoft Excel 2007 and Graph-Pad Prism v5.0. For real-time PCR assays, relative quantitation value of each miRNA was calculated by using the equation 2^−ΔΔCT^ and underwent log2-transformation to show the relatively expression levels of each target miRNAs. Statistical significance was determined using the Student’s *t* test. In all figures, values are expressed as mean ± standard deviation (SD), a *P* value <0.05 was considered to be statistically significant.

### Microarray Data Submission

The raw microarray data were submitted to Gene Expression Omnibus database, and is available under the following accession numbers GSE45816 and GSE45817.

## Results

### miRNA Profiling in HEV71-infected SH-SY5Y Cells

**Figure 1:**
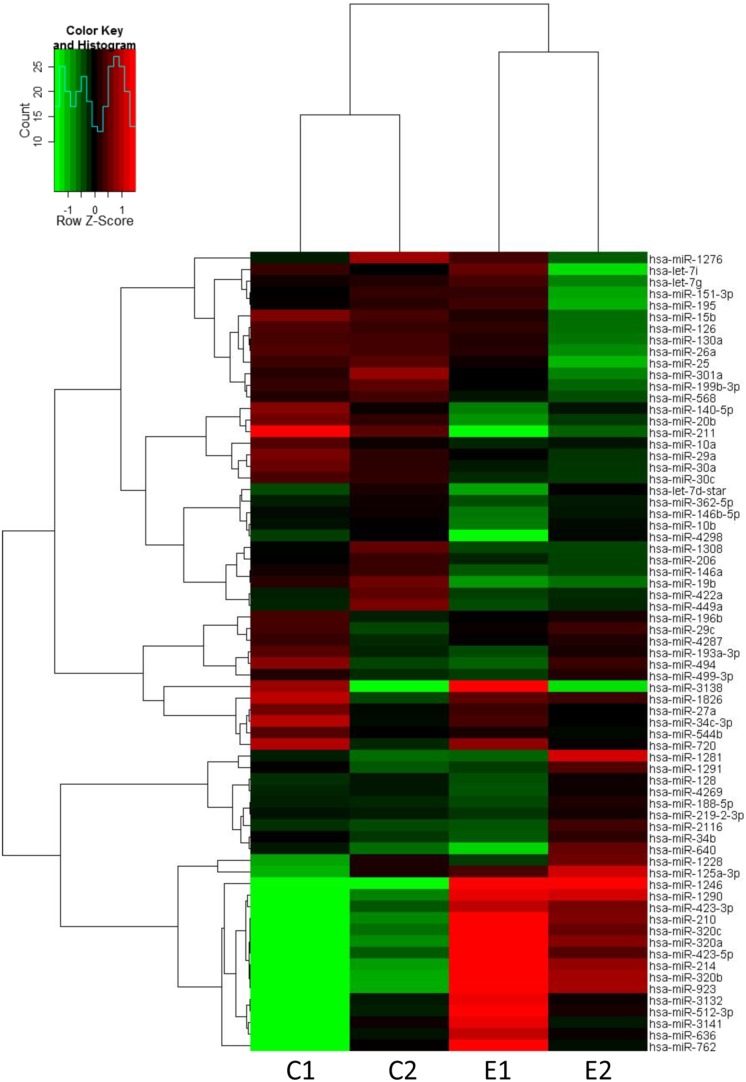
Heatmap and hierarchical clustering of miRNA. The heatmap represents the results of the two-way hierarchical clustering of miRNA and samples. Each row represents a miRNA, and each column represents a sample. C_1 and C_2 represent the non-infected cells at 6 and 12 hpi, respectively, and E_1 and E_2 represent the infected cells at 6 and 12 hpi, respectively. Up-regulated miRNAs are shown in red, and down-regulated miRNAs are shown in green.

The expression profiling of human-specific miRNAs was performed in HEV71-infected SH-SY5Y cells using the Affymetrix GeneChip microarray assay. SH-SY5Y cells were infected with HEV71 at an MOI of 1, and the infected cells were harvested after 6 and 12 hpi. The hierarchical clustering heatmap shows all differentially regulated miRNAs in the four independent samples according to the criteria of a fold change ≥1.5 or ≤−1.5 (*P*<0.05) at 6 (C1 and E1) and 12 (C2 and E2) hpi (**Fig. 1**). Further analysis showed that 69 miRNAs were differentially expressed during HEV71 infection compared with those in the uninfected cells (**Table S1**). Among the 69 identified miRNAs, miR-1246 exhibited significant 7- and 9-fold increases at 6and 12 hpi, respectively, compared with those in the uninfected cells.

**Figure 2:**
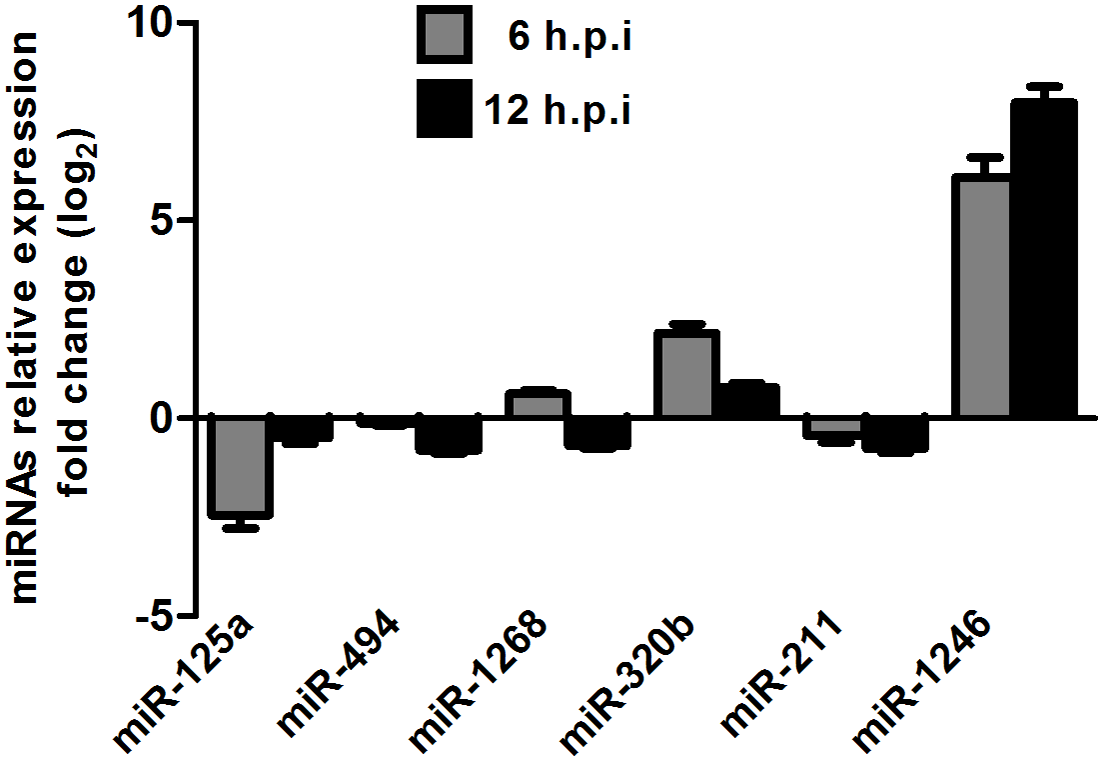
Validation of the microarray data using qRT-PCR. Selected miRNAs in HEV71-infected SH-SY5Y cells were validated using qRT-PCR. The gray bar and the black bar represent 6 and 12 hpi, respectively. The fold change was calculated based on endogenous control normalization using the equation 2^−ΔΔCt^. ***P<0.0001; values represent the mean±SD.

To validate the microarray results, six miRNAs were selected for independent qRT-PCR assays. As shown in **Fig. 2**, the expression levels of miR-125a significantly decreased, while miR-320b and miR-1246 significantly increased at 6 and 12 hpi; No significant change was observed for miR-1268 at either time points. These results were in agreement with the microarray analysis. These results strongly indicated that miR-1246 was significantly up-regulated in HEV71-infected SH-SY5Y cells.

### miR-1246 is Specifically Induced by Enterovirus Infection in SH-SY5Y Cells

**Figure 3:**
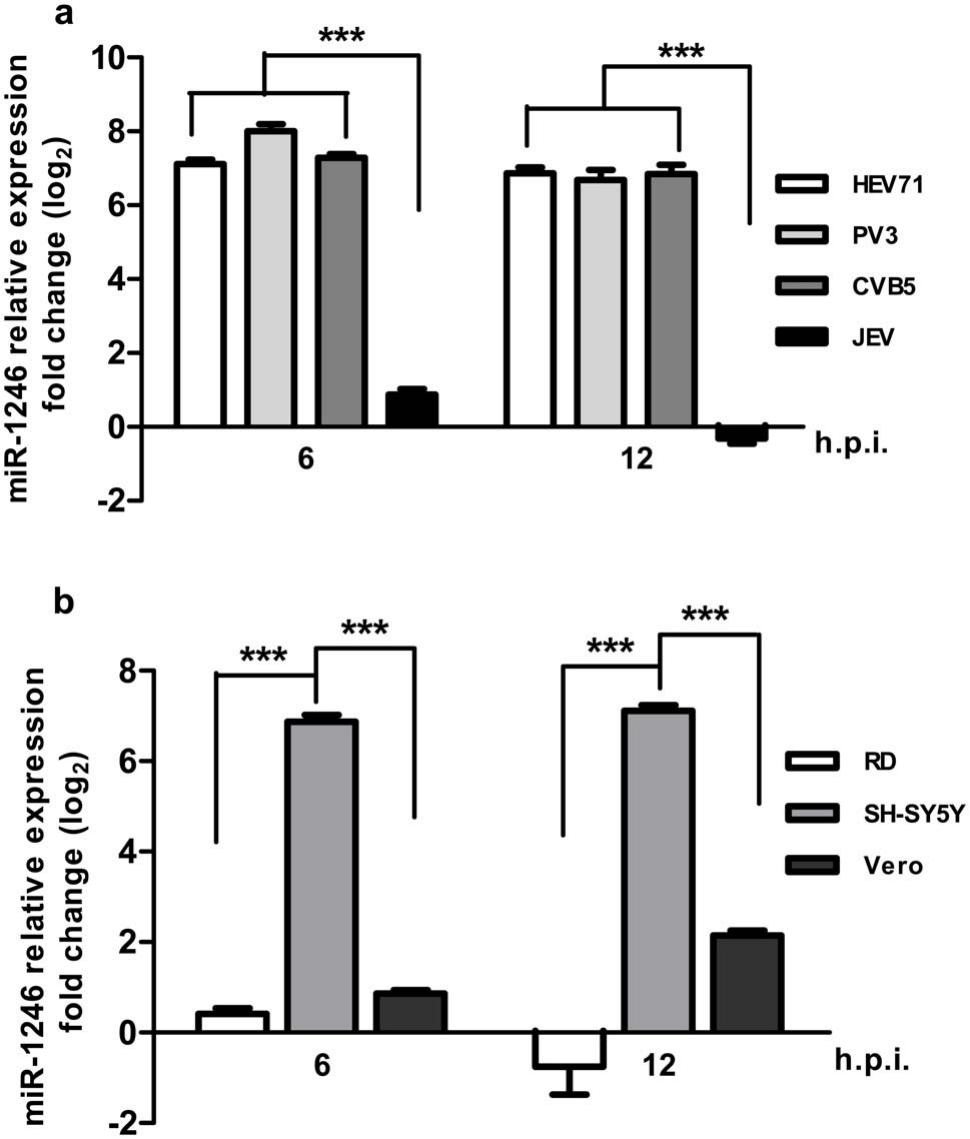
miR-1246 is specially induced in enterovirus-infected SH-SY5Y cells. **a.** SH-SY5Y cells were infected with HEV71, PV-3, CV-B5, and JEV, respectively, and the expression of miR-1246 was detected by qRT-PCR. **b.** SH-SY5Y cells, RD cells, and Vero cells were infected with HEV71 and subjected to qRT-PCR analysis for miR-1246. All data are normalized against a mock-infected sample. ***P<0.0001; values represent the mean±SD.

Previously, the expression of miR-1246 was identified in human embryonic stem cells [37]. To further characterize the expression pattern of miR-1246 in response to virus infection, SH-SY5Y cells were infected with various viruses, and the levels of miR-1246 were analyzed at 6 and 12 hpi. As shown in **Fig. 3A**, PV-3 and CV-B5 significantly induced the up-regulation of miR-1246 in SH-SY5Y cells, as did HEV71. However, no significant difference in miR-1246 levels was observed in the JEV-infected cells (**Fig. 3A**). Another two enterovirus-permissive cell lines, RD cells and Vero cells, were infected with HEV71, followed by miR-1246 assay at 6 and 12 hpi. The results showed that up-regulation of miR-1246 was only observed in HEV71-infected SH-SY5Y cells; no significant change was observed in RD cells, and only a slight change was observed in Vero cells at 12 hpi (**Fig. 3B**). Together, these results indicated that the up-regulation of miR-1246 is a specific response to HEV71 infection in human neuroblastoma cells.

### miR-1246 does not Affect HEV71 Replication

miRNAs can exert regulatory effects on both the host and the pathogen. To further investigate the potential effects of miR-1246 on viral RNA replication, SH-SY5Y cells were infected with HEV71 at an MOI of 1, following transfection with the miR-1246 inhibitor or the negative control. The miR-1246 inhibitor is a chemically synthetic oligonucleotide with a complete complementary sequence to endogenous miR-1246. At 6 and 12 hpi, viral titers in culture supernatants were then determined using qRT-PCR. As shown in **Fig. S1**, no significant difference was observed at 6 and 12 hpi with or without the miR-1246 inhibitor, indicating the inhibition of miR-1246 did not significantly affect HEV71 replication.

### Gene Expression Profiling and Target Predication

miRNAs regulate cellular gene expression at the post-transcriptional level, thus silencing and/or down-regulating gene expression. To assess whether a direct correlation exists between the expression patterns of mRNA and the target genes of miR-1246, we assessed the expression profile of the mRNA transcripts present in HEV71-infected SH-SY5Y cells, following transfection with the miR-1246 inhibitor, and control cells using a microarray assay based on the 35 K Human Genome Array. Microarray hybridization preliminarily identified mRNAs whose relative abundance increased or decreased during HEV71 infection, as shown in **Table S2** and **Table S3**. Among the 35035 transcripts tested (from human genome Oligo database), 182 genes were identified to be differentially regulated in the infected cells compared to the uninfected controls. Of the 182 differentially regulated genes, 97 genes increased in abundance with a fold change >1.5 (*P*<0.005), while 85 mRNAs decreased in abundance with a fold change<−1.5 (*P*<0.005).

**Figure 4:**
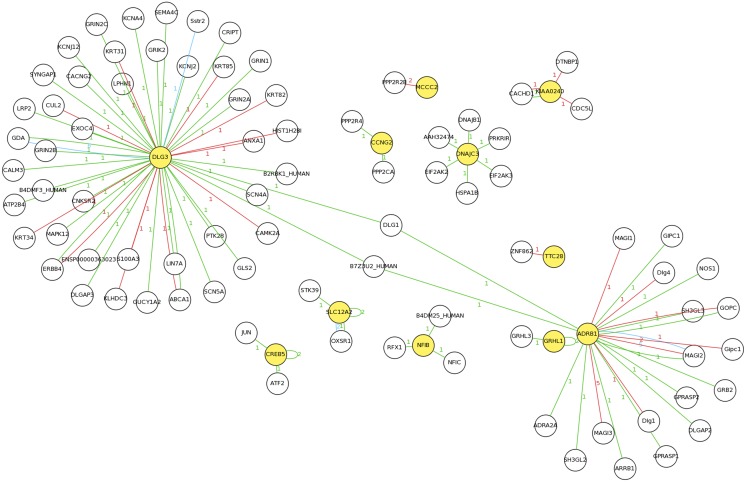
Predicted interaction networks of co-occurrence genes. Differentially regulated genes are represented in the links predicted using CMAS 3.0. Yellow sphere represents co-occurrence genes identified in our study, whereas white one represents genes form the database. The color of the lines represents methods to detect the white-labeled interactors of the yellow-labeled protein. Red line represents “anti bait coimmunoprecipitation”. Blue line represents “*in vitro*; *in vivo*; yeast 2-hybrid”. Green represents “*in vitro*; *in vivo*”. The number on top of the lines means MAS ID.

The potential target genes of miR-1246 were predicted using TargetScan6.0 online, and a total of 178 conserved target genes were shown in **Table S4**. To further analyze the potential correlation between the up-/down-regulated mRNAs and the predicted targets, a total of 14 co-occurrence genes were identified (**Table S5**). All these genes were then modeled into ontological networks using MAS3.0, and potential interaction is shown in **Fig. 4**. Among the 14 co-occurrence genes, four of them, VPS53, PSD3, C6orf35 and Cxorf36, were identified as orphan genes without interaction with any genes from the inclusive database.

**Figure 5:**
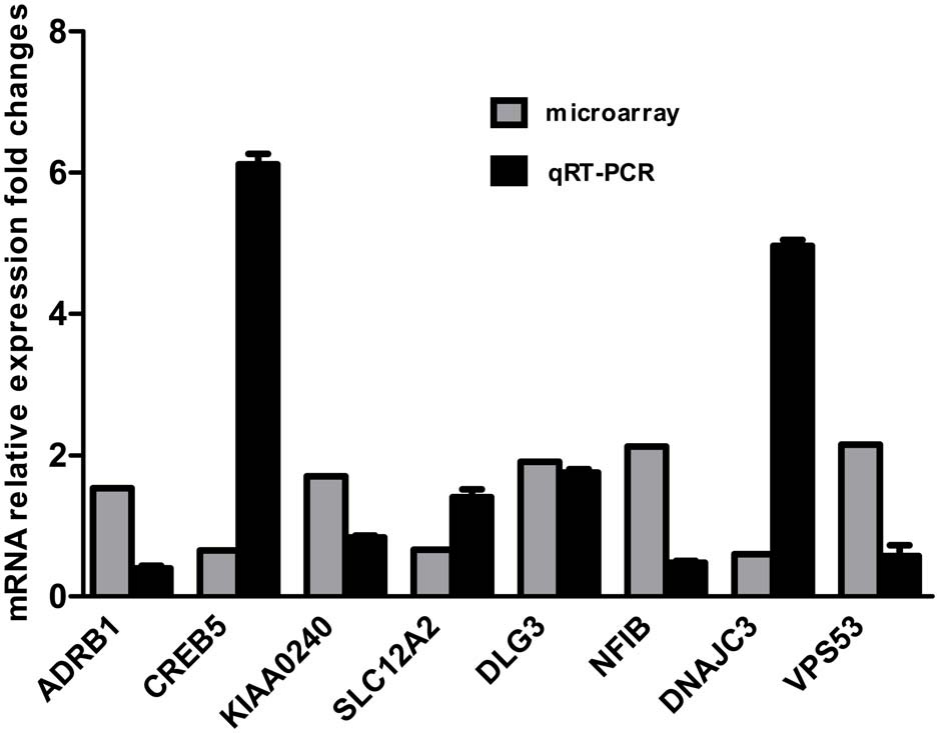
Validation of co-occurrence genes expression in HEV71-infected SH-SY5Y cells by qRT-PCR. A total of 14 co-occurrence genes were validated using real-time RT-PCR at 12 hpi. Relative fold change was calculated based on endogenous control normalization and repeated three times independently, and error bars present as mean±SD (n = 3). The results from qRT-PCR were compared with that from microarray.

Furthermore, all the co-occurrence genes were validated using qRT-PCR. As shown in **Fig. 5**, a total of eight genes, including ADRB1, CREB5, KIAA0240, SLC112A2, DSG3, NFIB, DNAJC3, and VPS53, were significantly induced by HEV71 infection, which was consistent with the mRNA microarray data. Finally, the DLG3 gene was selected for the following assays.

### miR-1246 Target the 3′-UTR of *DLG3*

**Figure 6:**
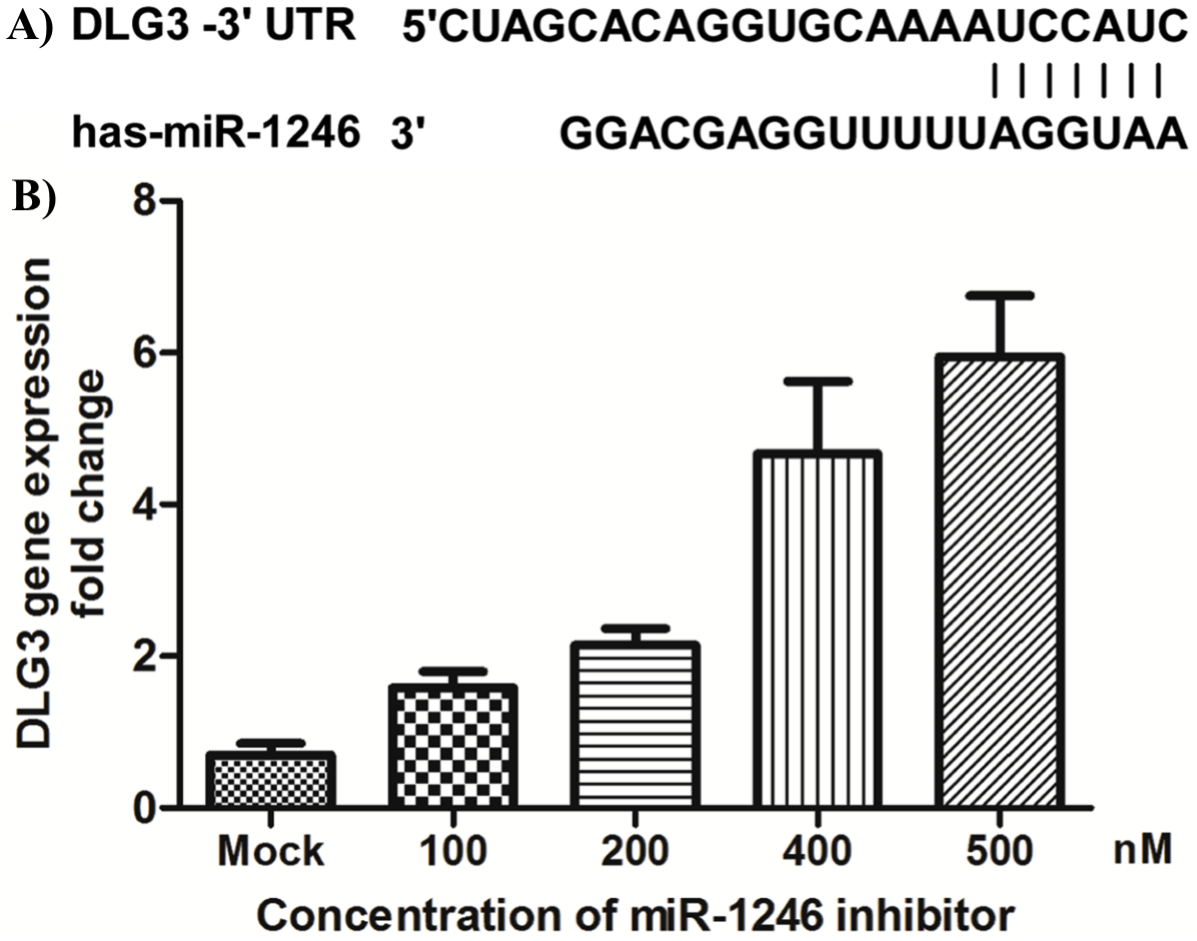
DLG3 is repressed by miR1246 in HEV71-infected SH-SY5Y cells. **A.** Predicted sequence of the miR-1246 binding sites within the *DLG3* 3′-UTR are indicated with vertical lines. **b.** *DLG3* gene expression in HEV71-infected SH-SY5Y cells was analyzed by qRT-PCR following transfection with varying doses of miR-1246 inhibitor. The data are normalized against mock transfection.

**Figure 7:**
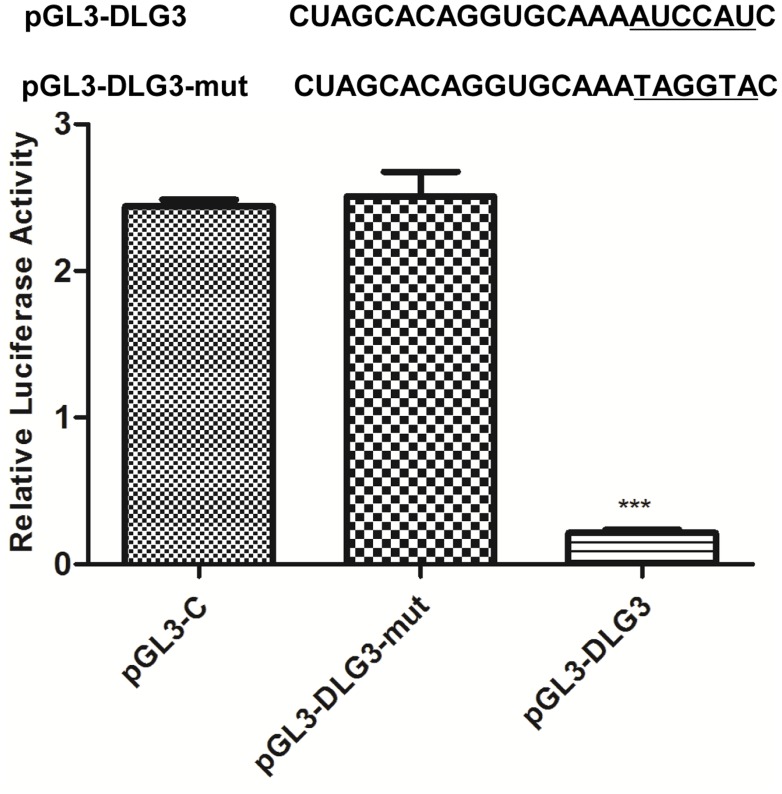
The 3′-UTR of DLG3 was the target of miR-1246. Reporter construct pLG3-DLG3-mut that destroyed the putative binding sites (underlined) is shown in comparison with wild type pLG3-DLG3 construct. The expression of pLG3-DLG3 was significantly decreased by miR-1246 in comparison with vector control and pLG3-DLG3-mut (***p<0.001). Each transfection was performed in triplicate.

To clarify the roles of host miR-1246 in HEV71 infection, miRanda and TargetScan databases were initially used to screen for potential targets of miR-1246 within HEV71 genome. The results showed that no miR-1246 complementary sequences were found in the HEV71 genome RNA transcripts. The potential miR-1246-binding sites within the 3′-UTR of *DLG3* were further predicted using TargetScan 6.0, and one specific miR-1246 binding site was accessed (**Fig. 6A**). Further, to test whether miR-1246 directly target the 3′-UTR of *DLG3*, dual luciferase reporter assay was performed in pS-miR-1246-transfected cells. As shown in Fig. 7, the expression of *DLG3* was significantly inhibited by miR-1246, while pGL3-DLG3-mut that destroyed the binding sites and vector control were not affected by miR1246 (p<0.001).

Finally, to clarify the relationship between miR-1246 and *DLG3* regulation in response to HEV71 infection, varying doses of miR-1246 inhibitor were transfected to inhibit the induction of miR-1246. The results showed that the suppression of *DLG3* caused by HEV71 infection was significantly eliminated by the transfection of miR-1246 inhibitor in dose-dependent manner (Fig. 6B). Together, all these results clearly demonstrated that the up-regulation of miR-1246 in response to HEV71 infection inhibited the expression of *DLG3*.

## Discussion

Human SH-SY5Y cells are the third successive subclone of the SK-N-SH (human neuroblastoma cell) cells [38]–[40], and these cells have been used to study HEV71 neural cell tropism [41]. In this study, the first miRNA profiling of SH-SY5Y cells infected with HEV71 was performed. Sixty-nine miRNAs were differentially regulated, suggesting that virus infection does alter the miRNA expression profile in SH-SY5Y cells. On the one hand, the HEV71-mediated miRNA expression was assessed in the SH-SY5Y cell line, as well as in different cell types. Interestingly, one of the 69 miRNAs, miR-1246, was expressed at significantly higher levels in SH-SY5Y cells than in RD or Vero cells. These results differ from a previous study that showed that miR-141 significantly increased in RD cells infected with enteroviruses [28]. It is not surprising because miRNAs play a major part in the tissue-specific regulation of gene expression patterns [42], [43], and several miRNAs have been shown to modulate the tissue tropism of a number of viruses from different families [44]–[47]. In addition, the special role ofmiR-1246 has been studied, especially on cancer and cystic fibrosis [48]–[51]. Thus, how miR-1246 interacts with HEV71 is of highly interests. Recent studies have reported that miRNAs play important roles in the host cellular response to viral infections [18], [21], [52], which can be attributed to both antiviral defenses and viral factors altering the cellular environment. Moreover, cellular or viral miRNAs have been shown to be involved in reciprocal interaction between the host cells and virus [25], [53]; for example, miR-122 treatment has been shown to enhance hepatitis C virus (HCV) replication by targeting the 5′-UTR [54]. However, our results showed that inhibition of miR-1246 did not affect HEV71 production at 6 and 12 hpi (**Fig. S1**). These results ruled out the possibility that up-regulation of miR-1246 may facilitate or suppress viral replication.

Alterations of the miRNA profile underlie global cell and tissue transcriptional changes during developmental processes and senescence, as well as during neoplastic transformation [55], [56]. Likewise, it has been shown that miRNAs are involved in the pathogenesis of the neurodegenerative disorders in multiple diseases, including AD and Parkinson’s disease [57], [58]. According to our results, HEV71 infection can disturb the expression of host miRNAs. It is well known that miRNAs can regulate post-transcriptional processes by binding to the 3′-UTR of the target transcript. miR-1246 has been shown to target the 3′-UTR sequence of the *DYRK1A* mRNA, resulting in a reduction in *DYRK1A* levels [59]; however, in our study, sequence analysis showed that this miRNA does not target the 3′-UTR or 5′-UTR of HEV71, which is in agreement with virus growth experiments (**Fig. S1**).

Precise identification of miRNA targets is necessary for the functional characterization of individual miRNAs and for a better understanding of complex human diseases. Gene expression profiling has been used to improve the target prediction for the identification of functional targets [60], [61]. A miRNA-mRNA regulatory module consists of a set of miRNAs and a set of their targets. One miRNA can potentially regulate multiple mRNAs, and the opposite is also possible [62]. Our results demonstrated that the 3′-UTR of *DLG3* gene were a potential target of miR-1246. DLG3 is the first XLMR gene and is linked directly to NMDA receptor-mediated signaling and synaptic plasticity [63], [64]. Compared with the classical transcription factors, miR-1246 directly targeted the 3′-UTR of *DLG3* mRNA (**Fig. 7**), and its expression was inversely correlated with *DLG3* expression in HEV71-infected SH-SY5Y cells (**Fig. 6**). In this study, we combined results from gene chips, mRNA assay, computational predictions, and dual luciferase assay to confirm miR-1246 directly targets *DLG3* in HEV71-infected neuroblastoma cells.

The predicted interaction networks of genes in **Fig. 4** show significant over representation of known mental disorder susceptibility genes [65], [66], supporting the increased power of the network-based approach in identifying disease-relevant transcriptional changes. *DLG3* is a membrane-associated guanylate kinase protein family (MAGUK) that is localized to the postsynaptic density of excitatory synapses [67]. Synapse-associated proteins are thought to have important functional roles within neuronal cells [68], [69]. Moreover, investigators reported that *DLG3* was part of a trafficking complex toward the synapse and was regulated by its PPxY motifs, which bind to the domain of Nedd4 and Nedd4-2 E3 ubiquitin ligases in neurons [70], [71]. *DLG3* has been demonstrated to control apical epithelial polarity and tight junction formation and to contribute to neural induction in mouse development [72]. Mutation of *DLG3* leads to synaptic dysfunction and learning and memory impairments [69], [73].

Dysregulation of synaptic plasticity has been involved in a variety of psychiatric disorders, neurological disorders, and in age-related cognitive impairment. Previous studies have reported that neurodevelopment and cognitive function may be affected by viral encephalitis or by bacterial meningitis [74]–[76]. Recently, Chang *et al.* showed that HEV71 infection with central nervous system (CNS) involvement and cardiopulmonary failure may be associated with neurologic sequelae, delayed neurodevelopment, and reduced cognitive functioning [77]. Considering the presence of HEV71 viral antigens in neurons [12], [13], direct HEV71 invasion and subsequent neuron damage may contribute to the main cause of neurologic sequelae. Our results set up connection between the neurologic sequelae caused by HEV71 infection and host miR-1246 regulation in association with *DLG3* gene.

In summary, this is the first report on whole-genome joint mRNA and miRNA profile analysis from EV71-infected SH-SY5Y cells. Among the 69 differentially expressed miRNAs, miR-1246 was expressed at significantly higher levels. The HEV71-mediated up-regulation of miR-1246 reduced the levels of DLG3 protein in SH-SY5Y cells. Together, these results indicate that miR-1246 play a potential role in the neurological process and cell death pathways by regulating *DLG3* upon HEV71 infection in human neuroblastoma cells.

## Supporting Information

Figure S1 HEV71 virus replication was uncorrelated to miR-1246 in SH-SY5Y cells.
(DOCX)

Table S1 Differentially expressed miRNAs in SH-SY5Y cells infected and non-infected with HEV71 virus by microarray assay.
(DOCX)

Table S2 Up-regulated genes in SH-SY5Y cells infected with HEV71 after transfection miR-1246 inhibitor by mRNA microarray assay.
(DOCX)

Table S3 Down-regulated genes in SH-SY5Y cells infected with HEV71 after transfection miR-1246 inhibitor by mRNA microarray assay.
(DOCX)

Table S4 The predicted miRNA targets of miR-1246 by Targetscan Human 6.0.
(DOCX)

Table S5 Co-occurrence genes and detection primers used for qRT-PCR analysis.
(DOCX)
