# Supplementary material for: Parallel mRNA and MicroRNA Profiling of HEV71-Infected Human Neuroblastoma Cells Reveal the Up-Regulation of miR-1246 in Association with DLG3 Repression
Source: PLoS One. 2014 Apr 16;9(4):e95272. doi: 10.1371/journal.pone.0095272 (PMC3989279; doi:10.1371/journal.pone.0095272)
Supplement: Figure S1 — HEV71 virus replication was uncorrelated to miR-1246 in SH-SY5Y cells. (DOCX) [file pone.0095272.s001.docx]

**Figure S1 HEV71 virus replication was uncorrelated to miR-1246 in SH-SY5Y cells.**

HEV71 virus yield was measured using qRT-PCR assay in HEV71-infected SH-SY5Y cells transfected with miR-1246 inhibitor or negative control (NC). All experiments were performed in triplicates and error bars present as mean ± SD (n=3).
