# Supplementary material for: Parallel mRNA and MicroRNA Profiling of HEV71-Infected Human Neuroblastoma Cells Reveal the Up-Regulation of miR-1246 in Association with DLG3 Repression
Source: PLoS One. 2014 Apr 16;9(4):e95272. doi: 10.1371/journal.pone.0095272 (PMC3989279; doi:10.1371/journal.pone.0095272)
Supplement: Table S1 — Differentially expressed miRNAs in SH-SY5Y cells infected and non-infected with HEV71 virus by microarray assay. (DOCX) [file pone.0095272.s002.docx]

**Table S1** Differentially expressed miRNAs in SH-SY5Y cells infected and non-infected with HEV71 virus by microarray assay

| miRNA | Ration1  (infected/non-infected 6h) | Ration2  (infected/non-infected 12h) | | position  on chromosomes |
| --- | --- | --- | --- | --- |
| hsa-miR-1246 | 7.856 | | 9.74681 | 2:9672371-9672474 (-) |
| hsa-miR-3138 | 3.22676 | | 1.11529 | 18:12533423-12533524 (-) |
| hsa-miR-762 | 2.17876 | | 0.8804 | 5:137896732-137896799 (-) |
| hsa-miR-320a | 1.9958 | | 1.36687 | 3:52722898-52722977 (+) |
| hsa-miR-320b | 1.90464 | | 1.49918 | 1:52190447-52190568 (-) |
| hsa-miR-923 | 1.87704 | | 1.50438 | 16:70571908-70572001 (+) |
| hsa-miR-1290 | 1.79763 | | 1.5204 | 16:28683554-28683657 (-) |
| hsa-miR-210 | 1.79192 | | 1.34367 | 1:224524493-224524591 (+) |
| hsa-miR-214 | 1.78849 | | 1.43906 | 8:59574800-59574903 (-) |
| hsa-miR-320c | 1.66897 | | 1.24753 | 1:52190447-52190568 (-) |
| hsa-miR-423-5p | 1.66674 | | 1.17799 | 6:128934346-128934452 (+) |
| hsa-miR-423-3p | 1.62288 | | 1.23704 | 12:57255159-57255292 (+) |
| hsa-miR-3141 | 1.61561 | | 0.85698 | 10:106159563-106159661 (+) |
| hsa-miR-512-3p | 1.57589 | | 0.98288 | 15:56247899-56248002 (-) |
| hsa-miR-636 | 1.54026 | | 0.94494 | 2:86362993-86363129 (+) |
| hsa-miR-3132 | 1.50647 | | 0.95947 | 3:53367087-53367155 (-) |
| hsa-miR-125a-3p | 1.02108 | | 1.19072 | 5:68471651-68471754 (-) |
| hsa-miR-1228 | 0.81825 | | 1.02917 | 22:21765339-21765415 (+) |
| hsa-miR-1276 | 0.80457 | | 0.62322 | 7:10262424-10262637 (+) |
| hsa-miR-195 | 0.75694 | | 0.65327 | 2:75645972-75646075 (-) |
| hsa-let-7g | 0.75374 | | 0.7087 | 18:53746625-53746825 (-) |
| hsa-let-7i | 0.75115 | | 0.64926 | 17:19565313-19565403 (+) |
| hsa-miR-151-3p | 0.7509 | | 0.6528 | 2:195528831-195528898 (-) |
| hsa-miR-126 | 0.66571 | | 0.70527 | 15:25467495-25467575 (+) |
| hsa-miR-422a | 0.66479 | | 0.73879 | 12:57255159-57255292 (+) |
| hsa-miR-128 | 0.66428 | | 0.95866 | 1:38349909-38349989 (+) |
| hsa-miR-219-2-3p | 0.66312 | | 0.97299 | 7:56123058-56123195 (+) |
| hsa-miR-130a | 0.66269 | | 0.68567 | 7:23934647-23934753 (-) |
| hsa-miR-206 | 0.66171 | | 0.75366 | 14:45036116-45036209 (-) |
| hsa-miR-2116 | 0.66146 | | 1.11687 | 8:103400754-103400863 (-) |
| hsa-miR-720 | 0.65948 | | 0.97247 | 4:152023209-152023283 (+) |
| hsa-miR-301a | 0.6588 | | 0.59489 | 6:4076623-4076707 (+) |
| hsa-miR-188-5p | 0.65577 | | 1.00639 | 13:106549872-106549992 (-) |
| hsa-miR-449a | 0.65576 | | 0.71391 | 12:119326239-119326369 (-) |
| hsa-miR-25 | 0.653 | | 0.60436 | 4:129677181-129677284 (+) |
| hsa-miR-26a | 0.65223 | | 0.66467 | 1:236464280-236464351 (+) |
| hsa-miR-4269 | 0.64983 | | 0.95685 | 3:8973179-8973316 (+) |
| hsa-miR-362-5p | 0.64828 | | 0.85134 | 1:110815106-110815229 (+) |
| hsa-miR-27a | 0.64619 | | 0.91849 | 1:54991059-54991126 (+) |
| hsa-miR-199b-3p | 0.64476 | | 0.67906 | 7:23436065-23436135 (+) |
| hsa-miR-568 | 0.64227 | | 0.73095 | 1:178722789-178722907 (-) |
| hsa-miR-4287 | 0.64163 | | 1.01392 | 18:9277977-9278077 (+) |
| hsa-miR-29c | 0.64082 | | 1.10604 | 6:74103636-74103736 (-) |
| hsa-miR-544b | 0.63692 | | 0.89534 | 8:142457575-142457649 (+) |
| hsa-miR-1291 | 0.63344 | | 1.17129 | 15:65989171-65989274 (-) |
| hsa-miR-1308 | 0.6296 | | 0.70765 | 2:227833705-227833838 (-) |
| hsa-miR-1281 | 0.62869 | | 1.47149 | 13:22101939-22102039 (-) |
| hsa-miR-196b | 0.6263 | | 0.98306 | 1:202200792-202200892 (+) |
| hsa-miR-34b | 0.60999 | | 1.0541 | 8:92476263-92476367 (-) |
| hsa-let-7d-star | 0.60855 | | 0.87429 | 20:37070679-37070812 (+) |
| hsa-miR-1826 | 0.60444 | | 1.099 | 7:138167225-138167328 (-) |
| hsa-miR-499-3p | 0.60362 | | 1.01774 | 1:12281205-12281328 (-) |
| hsa-miR-15b | 0.60267 | | 0.6913 | 2:3628160-3628322 (+) |
| hsa-miR-146a | 0.59402 | | 0.75422 | 3:45296902-45297112 (-) |
| hsa-miR-10b | 0.59283 | | 0.88878 | 11:116191061-116191162 (+) |
| hsa-miR-146b-5p | 0.59172 | | 0.85947 | 15:65863311-65863411 (-) |
| hsa-miR-34c-3p | 0.58791 | | 0.9235 | 5:139906937-139907013 (-) |
| hsa-miR-30c | 0.58506 | | 0.77703 | 1:154260936-154261003 (+) |
| hsa-miR-10a | 0.58333 | | 0.86873 | 1:165041710-165041781 (-) |
| hsa-miR-29a | 0.5819 | | 0.78769 | 1:86057963-86058039 (+) |
| hsa-miR-30a | 0.57416 | | 0.78151 | 18:19291911-19292080 (-) |
| hsa-miR-193a-3p | 0.54946 | | 0.99155 | 4:108038489-108038561 (-) |
| hsa-miR-640 | 0.52505 | | 1.24455 | 2:203141154-203141241 (+) |
| hsa-miR-19b | 0.52118 | | 0.64917 | 3:121310141-121310241 (+) |
| hsa-miR-4298 | 0.51436 | | 0.89864 | 7:52391494-52391585 (-) |
| hsa-miR-494 | 0.49049 | | 1.09593 | 6:42473238-42473344 (-) |
| hsa-miR-20b | 0.47091 | | 0.77345 | 12:93659793-93659872 (+) |
| hsa-miR-140-5p | 0.47069 | | 0.86642 | 7:115221369-115221496 (-) |
| hsa-miR-211 | 0.22337 | | 0.68604 | 2:122463543-122463633 (-) |
