# Supplementary material for: Parallel mRNA and MicroRNA Profiling of HEV71-Infected Human Neuroblastoma Cells Reveal the Up-Regulation of miR-1246 in Association with DLG3 Repression
Source: PLoS One. 2014 Apr 16;9(4):e95272. doi: 10.1371/journal.pone.0095272 (PMC3989279; doi:10.1371/journal.pone.0095272)
Supplement: Table S2 — Up-regulated genes in SH-SY5Y cells infected with HEV71 after transfection miR-1246 inhibitor by mRNA microarray assay. (DOCX) [file pone.0095272.s003.docx]

**Table S2** Up-regulated genes in SH-SY5Y cells infected with HEV71 after transfection miR-1246 inhibitor by mRNA microarray assay.

| **gene_symbol** | **Description** | **Gene Bank** | **Fold change** |
| --- | --- | --- | --- |
| KCNE4 | Potassium voltage-gated channel subfamily E member 4 | NM_080671 | 35.2975 |
| RPRML | reprimo-like | NM_203400 | 22.5024 |
| COG6 | Conserved oligomeric Golgi complex component 6 | NM_020751 | 20.5763 |
| ANKRD11 | Ankyrin repeat domain-containing protein 11 | NM_013275 | 18.0199 |
| ZFYVE19 | Zinc finger FYVE domain-containing protein 19 | NM_032850 | 15.1892 |
| IREB2 | Iron-responsive element-binding protein 2 | NM_004136 | 14.0972 |
| C16orf76 | C16orf76 protein | NM_152339 | 11.2051 |
| FOS | Proto-oncogene protein c-fos | NM_005252 | 10.4273 |
| ZSCAN22 | GLI-Kruppel family member HKR2 | NM_181846 | 9.5064 |
| PIK3R4 | Phosphoinositide 3-kinase regulatory subunit 4 | NM_014602 | 8.9334 |
| KIRREL | Kin of IRRE-like protein 1 precursor | NM_018240 | 8.8314 |
| CASR | Extracellular calcium-sensing receptor precursor | NM_000388 | 7.7182 |
| EPM2A | Laforin | NM_005670 | 6.9764 |
| BPNT1 | 3'(2'),5'-bisphosphate nucleotidase 1 | NM_006085 | 6.7109 |
| RABEPK | Rab9 effector p40 | NM_005833 | 6.5090 |
| MTMR15 | myotubularin related protein 15 | NM_014967 | 6.4287 |
| EHD1 | EH domain-containing protein 1 | NM_006795 | 6.0577 |
| SLC27A2 | Very-long-chain acyl-CoA synthetase | NM_003645 | 5.8914 |
| DLX4 | Homeobox protein DLX-4 | NM_001934 | 5.8480 |
| WDR47 | WD repeat protein 47 | NM_014969 | 5.7597 |
| C20orf77 | Uncharacterized protein C20orf77 | NM_021215 | 5.6468 |
| KLHL17 | Kelch-like protein 17 (Actinfilin) | NM_198317 | 5.6397 |
| ZNF598 | Zinc finger protein 598 | NM_178167 | 5.4680 |
| IRF7 | Interferon regulatory factor 7 | NM_004031;NM_004030;  NM_004029;NM_001572 | 5.3243 |
| GLS2 | Glutaminase liver isoform, mitochondrial precursor | NM_013267 | 5.2982 |
| OR5AU1 | Olfactory receptor 5AU1 | NM_001004731 | 5.1560 |
| SLC35B4 | Solute carrier family 35 member B4 | NM_032826 | 5.1313 |
| VPS33A | Vacuolar protein sorting-associated protein 33A | NM_022916 | 5.0979 |
| CDKN1B | Cyclin-dependent kinase inhibitor 1B | NM_004064 | 4.9662 |
| DHDDS | Dehydrodolichyldiphosphate synthase | NM_205861;NM_024887 | 4.7636 |
| COL4A3BP | Goodpasture antigen-binding protein | NM_031361;NM_005713 | 4.7367 |
| UBE3B | ubiquitin protein ligase E3B | NM_183415;NM_183414;  NM_130466 | 4.4705 |
| KIAA0195 | KIAA0195 (KIAA0195), mRNA | NM_014738 | 4.3491 |
| RUSC1 | RUN and SH3 domain-containing protein 1 | NM_014328 | 4.3183 |
| STAT3 | Signal transducer and activator of transcription 3 | NM_003150;NM_139276;  NM_213662 | 4.2156 |
| PKP2 | Plakophilin-2 | NM_001005242;  NM_004572 | 4.0528 |
| DLX2 | Homeobox protein DLX-2 | NM_004405 | 4.0007 |
| RAD9A | Cell cycle checkpoint control protein RAD9A | NM_004584 | 3.9174 |
| ANGPTL6 | Angiopoietin-related protein 6 precursor | NM_031917 | 3.8717 |
| RBM12B | RNA-binding protein 12B | NM_203390 | 3.7763 |
| ARID3B | AT rich interactive domain 3B | NM_006465 | 3.6528 |
| ITGB5 | Integrin beta-5 precursor | NM_002213 | 3.6446 |
| RPAIN | RPA-interacting protein (hRIP) | NM_032308 | 3.6015 |
| INSM1 | Insulinoma-associated protein 1 | NM_002196 | 3.5788 |
| TAF3 | Transcription initiation factor TFIID subunit 3 | XM_291729 | 3.5745 |
| PRAMEF4 | PRAME family member 4 | XM_291396 | 3.5682 |
| ECEL1 | Endothelin-converting enzyme-like 1 | NM_004826 | 3.5496 |
| LGALS14 | Placental protein 13-like | NM_203471;NM_020129 | 3.3156 |
| CRB3 | Crumbs protein homolog 3 precursor | NM_174882;NM_139161;  NM_174881 | 3.3125 |
| PRCP | Lysosomal Pro-X carboxypeptidase precursor | NM_199418;NM_005040 | 3.2941 |
| PTGIS | Prostacyclin synthase | NM_000961 | 3.2806 |
| NUDT6 | Nucleoside diphosphate-linked moiety X motif 6 | NM_007083;NM_198041 | 3.0843 |
| SMURF2 | Smadubiquitination regulatory factor 2 | NM_022739 | 3.0814 |
| GREM1 | Gremlin-1 precursor | NM_013372 | 3.0521 |
| CNOT4 | CCR4-NOT transcription complex subunit 4 | NM_001008225;  NM_013316 | 2.9586 |
| SLC22A18 | Organic cation transporter-like protein 2 | NM_183233 | 2.9318 |
| C20orf23 | Kinesin-like motor protein C20orf23 | NM_024704 | 2.8643 |
| MGST2 | Microsomal glutathione S-transferase 2 | NM_002413;NM_002413 | 2.8423 |
| PHGDH | D-3-phosphoglycerate dehydrogenase | NM_006623 | 2.8145 |
| ITGB7 | Integrin beta-7 precursor | NM_000889 | 2.8022 |
| SGK | Serine/threonine-protein kinase Sgk1 | NM_005627 | 2.7923 |
| DHRS3 | Short-chain dehydrogenase/reductase 3 | NM_004753 | 2.6460 |
| CREB1 | cAMP response element-binding protein | NM_134442;NM_004379 | 2.6409 |
| ZFYVE9 | Zinc finger FYVE domain-containing protein 9 | NM_004799 | 2.5792 |
| PCP2 | Purkinje cell protein 2 homolog | XM_058956 | 2.5357 |
| AR | Androgen receptor | NM_000044 | 2.4515 |
| PHF8 | PHD finger protein 8 | NM_015107 | 2.4463 |
| SYN2 | Synapsin-2 | NM_133625;NM_003178 | 2.4353 |
| GOSR1 | Golgi SNAP receptor complex member 1 | NM_001007025;  NM_004871;  NM_001007024 | 2.4351 |
| ERBB2IP | Protein LAP2 (Erbb2-interacting protein) | NM_018695;  NM_001006600 | 2.3331 |
| GPM6B | Neuronal membrane glycoprotein M6-b | NM_001001995;  NM_005278;  NM_001001994;  NM_001001996 | 2.3288 |
| CDC27 | Cell division cycle protein 27 homolog | NM_001256 | 2.3236 |
| SLC25A40 | mitochondrial carrier family protein | NM_018843 | 2.3224 |
| C20orf39 | UPF0338 protein C20orf39 | NM_024893 | 2.2523 |
| STOX2 | storkhead box 2 | NM_020225 | 2.2490 |
| NARG2 | NMDA receptor regulated 2 isoform a | NM_024611 | 2.2487 |
| SLK | STE20-like serine/threonine-protein kinase | NM_014720 | 2.1906 |
| GRAMD1B | GRAMD1B protein | XM_370660 | 2.1840 |
| SLC3A2 | 4F2 cell-surface antigen heavy chain | NM_002394 | 2.1604 |
| ING5 | Inhibitor of growth protein 5 | XM_496035 | 2.1587 |
| SPRY3 | Sprouty homolog 3 | NM_005840 | 2.1546 |
| VPS53 | Vacuolar protein sorting-associated protein 53 | NM_018289.2 | 2.1543 |
| CXorf38 | Uncharacterized protein CXorf38 | NM_144970 | 2.1540 |
| NFIB | Nuclear factor 1 B-type | NM_005596 | 2.1243 |
| FGF22 | Fibroblast growth factor 22 precursor | NM_020637 | 2.1166 |
| SMEK1 | SMEK homolog 1 | NM_017936;NM_032560 | 2.1068 |
| EYA2 | Eyes absent homolog 2 | NM_172110;NM_172111;NM_172113;NM_172112;NM_005244 | 2.1052 |
| SLC9A8 | Sodium/hydrogen exchanger 8 | NM_015266 | 2.0529 |
| MLN | Motilin precursor | NM_002418 | 2.0370 |
| NTRK3 | NT-3 growth factor receptor precursor | NM_001007155;  NM_002530 | 2.0086 |
| ARIH2 | Protein ariadne-2 homolog | NM_006321 | 2.0069 |
| MSI2 | RNA-binding protein Musashi homolog 2 | NM_138962 | 2.0039 |
| DLG3 | Discs large homolog 3 | NM_021120.2;  NM_020730.1 | 1.9067 |
| GRHL1 | Grainyhead-like protein 1 homolog | NM_014552;NM_198182 | 1.8681 |
| KIAA0240 | KIAA0240 (KIAA0240), mRNA | NM_015349 | 1.7010 |
| CCNG2 | Cyclin-G2 | NM_004354 | 1.6748 |
| ADRB1 | Beta-1 adrenergic receptor | NM_000684 | 1.5324 |
