# Supplementary material for: Parallel mRNA and MicroRNA Profiling of HEV71-Infected Human Neuroblastoma Cells Reveal the Up-Regulation of miR-1246 in Association with DLG3 Repression
Source: PLoS One. 2014 Apr 16;9(4):e95272. doi: 10.1371/journal.pone.0095272 (PMC3989279; doi:10.1371/journal.pone.0095272)
Supplement: Table S3 — Down-regulated genes in SH-SY5Y cells infected with HEV71 after transfection miR-1246 inhibitor by mRNA microarray assay. (DOCX) [file pone.0095272.s004.docx]

**Table S3** Down-regulated genes in SH-SY5Y cells infected with HEV71 after transfection miR-1246 inhibitor (Ratio > 1.5, P < 0.05) by mRNA microarray assay.

| **gene_symbol** | **Description** | **Gene Bank** | **Fold change** |
| --- | --- | --- | --- |
| SLC12A2 | Solute carrier family 12 member 2 | NM_001046 | 0.6602 |
| CREB5 | cAMP response element-binding protein 5 | NM_004904;NM_182899;  NM_182898 | 0.6522 |
| C6orf35 | Uncharacterized bone marrow protein BM033 | NM_018452 | 0.6107 |
| DNAJC3 | DnaJ homolog subfamily C member 3 | NM_006260 | 0.5999 |
| MCCC2 | Methylcrotonoyl-CoA carboxylase beta chain | NM_022132 | 0.5669 |
| CXorf36 | Uncharacterized protein CXorf36 precursor | NM_024689 | 0.4987 |
| PSD3 | PH and SEC7 domain-containing protein 3 | NM_206909 | 0.4264 |
| SYT15 | Synaptotagmin-15 | NM_031912;NM_181519 | 0.4248 |
| TXNRD3 | TXNRD3 protein (Fragment) | XM_051264 | 0.4245 |
| AMICA1 | Junctional adhesion molecule-like precurso | NM_153206 | 0.4242 |
| TNS3 | tensin-like SH2 domain containing 1 | NM_022748 | 0.4189 |
| SULT1C1 | Sulfotransferase 1C1 | NM_001056;NM_176825 | 0.4182 |
| SDK2 | Protein sidekick-2 precursor | NM_019064 | 0.4117 |
| PIM3 | Serine/threonine-protein kinase Pim-3 | NM_001001852 | 0.4116 |
| LRRC27 | Leucine-rich repeat-containing protein 27 | NM_030626;XM_498500 | 0.4093 |
| PES1 | Pescadillo homolog 1 | NM_014303 | 0.4089 |
| GRM3 | Metabotropic glutamate receptor 3 precursor | NM_000840 | 0.4065 |
| TGFA | Transforming growth factor alpha precursor | NM_003236 | 0.4061 |
| RBM41 | RNA-binding protein 41 | NM_018301 | 0.3806 |
| DAK | Dihydroxyacetone kinase | NM_015533 | 0.3798 |
| CTNNAL1 | Alpha-catulin | NM_003798 | 0.3729 |
| PIK3AP1 | phosphoinositide-3-kinase adaptor protein 1 | NM_152309 | 0.3721 |
| RPL38 | 60S ribosomal protein L38 | NM_000999 | 0.3697 |
| SLC36A4 | solute carrier family 36 | NM_152313 | 0.3615 |
| KIAA1274 | KIAA1274 | NM_014431 | 0.3609 |
| AMFR | Autocrine motility factor receptor, isoform 2 | NM_138958;NM_001144 | 0.3577 |
| BICD1 | Protein bicaudal D homolog 1 | NM_001714 | 0.3506 |
| GGT1 | Gamma-glutamyltranspeptidase 1 precursor | NM_013421;NM_013430;  NM_005265 | 0.3452 |
| UPP1 | Uridinephosphorylase 1 | NM_003364;NM_181597 | 0.3428 |
| INSL4 | Early placenta insulin-like peptide precursor | NM_002195 | 0.3412 |
| KLC2 | Kinesin light chain 2 | NM_022822 | 0.3401 |
| SNW1 | SNW domain-containing protein 1 | NM_012245 | 0.3369 |
| MMP12 | Macrophage metalloelastase precursor | NM_002426 | 0.3263 |
| IDH3B | Isocitrate dehydrogenase [NAD] subunit beta, | NM_006899;NM_174855;  NM_174856 | 0.3203 |
| APOBEC1 | C->U-editing enzyme APOBEC-1 | NM_005889;NM_001644 | 0.3175 |
| FBP1 | Fructose-1,6-bisphosphatase 1 | NM_000507 | 0.3174 |
| CATSPER2 | sperm-associated cation channel 2 isoform 1 | NM_172096;NM_172097;  NM_054020;NM_172095 | 0.3162 |
| TEKT1 | Tektin-1 | NM_053285 | 0.3115 |
| DST | Bullous pemphigoid antigen 1, Dystonin | NM_001723 | 0.3096 |
| CELSR3 | SH3 adapter protein SPIN90 | NM_184231;NM_016453 | 0.3095 |
| GBA2 | bile acid beta-glucosidase | NM_020944 | 0.3014 |
| SPAG6 | Sperm-associated antigen 6 | NM_172242;NM_012443 | 0.3004 |
| INS | Insulin precursor | NM_000207 | 0.2996 |
| BTN3A1 | Butyrophilin subfamily 3 member A1 precursor | NM_194441;NM_007048 | 0.2993 |
| MAGEE2 | Melanoma-associated antigen E2 | NM_138703 | 0.2964 |
| CLDN11 | Claudin-11 | NM_005602 | 0.2930 |
| ABHD11 | abhydrolase domain containing 11 isoform 1 | NM_148916;NM_148913;  NM_148912;NM_148914;  NM_031295;NM_148915 | 0.2839 |
| CRYBA1 | Beta crystallin A3 | NM_005208 | 0.2833 |
| C20orf177 | Uncharacterized protein C20orf177 | NM_022106 | 0.2826 |
| APOC3 | Apolipoprotein C-III precursor | NM_000040;XM_496537 | 0.2798 |
| APRV1_HUMAN | Retroviral-like aspartic protease 1 precursor | NM_152792 | 0.2732 |
| TYR | Tyrosinase precursor | NM_000372 | 0.2727 |
| MAN2C1 | Alpha-mannosidase 2C1 | NM_006715 | 0.2694 |
| CDK2AP2 | Cyclin-dependent kinase 2-associated protein 2 | NM_005851 | 0.2677 |
| ISL1 | Insulin gene enhancer protein ISL-1 | NM_002202 | 0.2522 |
| F7 | Coagulation factor VII precursor | NM_000131;NM_019616 | 0.2519 |
| BMS1L | Ribosome biogenesis protein BMS1 homolog | XM_496031;XM_375148;  NM_080926;NM_014753;  XM_497500 | 0.2505 |
| DGKA | Diacylglycerol kinase alpha | NM_201445;NM_201554;  NM_001345;NM_201444 | 0.2448 |
| NELL1 | Protein kinase C-binding protein NELL1 precursor | NM_006157 | 0.2430 |
| HTR2B | 5-hydroxytryptamine 2B receptor | NM_000867 | 0.2355 |
| FOXD2 | Forkhead box protein D2 | NM_004474 | 0.2344 |
| C3orf64 | AER61 glycosyltransferase | NM_173654 | 0.2088 |
| KIAA0515 | CDNA: FLJ22509 fis, clone HRC11803 | XM_497080 | 0.2036 |
| GOLGA5 | Golgin subfamily A member 5 | NM_005113 | 0.2022 |
| ZFP36L2 | Butyrate response factor 2 | NM_006887 | 0.2004 |
| PRDM12 | PR domain zinc finger protein 12 | NM_021619 | 0.1989 |
| SPI1 | Transcription factor PU.1 | NM_003120 | 0.1967 |
| ACTL7B | Actin-like protein 7B | NM_006686 | 0.1923 |
| FAM102A | Protein FAM102A | NM_203305 | 0.1894 |
| HSD17B3 | Estradiol 17-beta-dehydrogenase 3 | NM_000197 | 0.1887 |
| SH3GL3 | SH3-containing GRB2-like protein 3 | NM_003027 | 0.1751 |
| SLC35E2 | solute carrier family 35, member E2 | NM_014854 | 0.1613 |
| C11orf76 | Putative uncharacterized protein C11orf76 | NM_145308 | 0.1609 |
| PCGF6 | Polycomb group RING finger protein 6 | NM_032154 | 0.1585 |
| PLS1 | Plastin-1 | NM_002670 | 0.1557 |
| TBX18 | T-box transcription factor TBX18 | XM_496819 | 0.1490 |
| HMGB3 | High mobility group protein B3 | XM_498174;XM_499404 | 0.1446 |
| C20orf59 | Putative transporter C20orf59 | NM_022082 | 0.1418 |
| HIST1H2BG | Histone H2B type 1-C/E/F/G/I | NM_003518 | 0.1383 |
| TWISTNB | TWIST neighbor | NM_001002926 | 0.1343 |
| MYH1 | Myosin-1 | NM_005963 | 0.1229 |
| KCNK10 | Potassium channel subfamily K member 10 | NM_021161;NM_138318;  NM_138317 | 0.1097 |
| CEP164 | centrosomal protein 164kDa | NM_014956 | 0.1030 |
| POU2AF1 | POU domain class 2-associating factor 1 | NM_006235 | 0.0930 |
| DGAT2 | Diacylglycerol O-acyltransferase 2 | NM_032564 | 0.0710 |
