# Supplementary material for: Parallel mRNA and MicroRNA Profiling of HEV71-Infected Human Neuroblastoma Cells Reveal the Up-Regulation of miR-1246 in Association with DLG3 Repression
Source: PLoS One. 2014 Apr 16;9(4):e95272. doi: 10.1371/journal.pone.0095272 (PMC3989279; doi:10.1371/journal.pone.0095272)
Supplement: Table S4 — The predicted miRNA targets of miR-1246 by Targetscan Human 6.0. (DOCX) [file pone.0095272.s005.docx]

**Table S4** The predicted miRNA targets of miR-1246 by Targetscan Human 6.0:

| Target gene | Representative transcript | Gene name | Links to sites in UTRs |
| --- | --- | --- | --- |
|  |  |  |  |
| [FAM53C](http://www.ncbi.nlm.nih.gov/sites/entrez?Db=gene&Cmd=ShowDetailView&TermToSearch=51307) | [NM_001135647](http://www.ncbi.nlm.nih.gov/entrez/query.fcgi?cmd=Search&db=nuccore&term==NM_001135647) | family with sequence similarity 53, member C | [Sites in UTR](http://www.targetscan.org/cgi-bin/targetscan/vert_60/view_gene.cgi?taxid=9606&rs=NM_001135647&members=miR-1246&showcnc=1&shownc=1&showncf=1) |
| [CREBL2](http://www.ncbi.nlm.nih.gov/sites/entrez?Db=gene&Cmd=ShowDetailView&TermToSearch=1389) | [NM_001310](http://www.ncbi.nlm.nih.gov/entrez/query.fcgi?cmd=Search&db=nuccore&term==NM_001310) | cAMP responsive element binding protein-like 2 | [Sites in UTR](http://www.targetscan.org/cgi-bin/targetscan/vert_60/view_gene.cgi?taxid=9606&rs=NM_001310&members=miR-1246&showcnc=1&shownc=1&showncf=1) |
| [ANTXR2](http://www.ncbi.nlm.nih.gov/sites/entrez?Db=gene&Cmd=ShowDetailView&TermToSearch=118429) | [NM_058172](http://www.ncbi.nlm.nih.gov/entrez/query.fcgi?cmd=Search&db=nuccore&term==NM_058172) | anthrax toxin receptor 2 | [Sites in UTR](http://www.targetscan.org/cgi-bin/targetscan/vert_60/view_gene.cgi?taxid=9606&rs=NM_058172&members=miR-1246&showcnc=1&shownc=1&showncf=1) |
| [RTKN2](http://www.ncbi.nlm.nih.gov/sites/entrez?Db=gene&Cmd=ShowDetailView&TermToSearch=219790) | [NM_145307](http://www.ncbi.nlm.nih.gov/entrez/query.fcgi?cmd=Search&db=nuccore&term==NM_145307) | rhotekin 2 | [Sites in UTR](http://www.targetscan.org/cgi-bin/targetscan/vert_60/view_gene.cgi?taxid=9606&rs=NM_145307&members=miR-1246&showcnc=1&shownc=1&showncf=1) |
| [REPS2](http://www.ncbi.nlm.nih.gov/sites/entrez?Db=gene&Cmd=ShowDetailView&TermToSearch=9185) | [NM_001080975](http://www.ncbi.nlm.nih.gov/entrez/query.fcgi?cmd=Search&db=nuccore&term==NM_001080975) | RALBP1 associated Eps domain containing 2 | [Sites in UTR](http://www.targetscan.org/cgi-bin/targetscan/vert_60/view_gene.cgi?taxid=9606&rs=NM_001080975&members=miR-1246&showcnc=1&shownc=1&showncf=1) |
| [ZNF697](http://www.ncbi.nlm.nih.gov/sites/entrez?Db=gene&Cmd=ShowDetailView&TermToSearch=90874) | [NM_001080470](http://www.ncbi.nlm.nih.gov/entrez/query.fcgi?cmd=Search&db=nuccore&term==NM_001080470) | zinc finger protein 697 | [Sites in UTR](http://www.targetscan.org/cgi-bin/targetscan/vert_60/view_gene.cgi?taxid=9606&rs=NM_001080470&members=miR-1246&showcnc=1&shownc=1&showncf=1) |
| [HS3ST1](http://www.ncbi.nlm.nih.gov/sites/entrez?Db=gene&Cmd=ShowDetailView&TermToSearch=9957) | [NM_005114](http://www.ncbi.nlm.nih.gov/entrez/query.fcgi?cmd=Search&db=nuccore&term==NM_005114) | heparan sulfate (glucosamine) 3-O-sulfotransferase 1 | [Sites in UTR](http://www.targetscan.org/cgi-bin/targetscan/vert_60/view_gene.cgi?taxid=9606&rs=NM_005114&members=miR-1246&showcnc=1&shownc=1&showncf=1) |
| [ZNF323](http://www.ncbi.nlm.nih.gov/sites/entrez?Db=gene&Cmd=ShowDetailView&TermToSearch=64288) | [NM_001135215](http://www.ncbi.nlm.nih.gov/entrez/query.fcgi?cmd=Search&db=nuccore&term==NM_001135215) | zinc finger protein 323 | [Sites in UTR](http://www.targetscan.org/cgi-bin/targetscan/vert_60/view_gene.cgi?taxid=9606&rs=NM_001135215&members=miR-1246&showcnc=1&shownc=1&showncf=1) |
| [MCCC2](http://www.ncbi.nlm.nih.gov/sites/entrez?Db=gene&Cmd=ShowDetailView&TermToSearch=64087) | [NM_022132](http://www.ncbi.nlm.nih.gov/entrez/query.fcgi?cmd=Search&db=nuccore&term==NM_022132) | methylcrotonoyl-CoA carboxylase 2 (beta) | [Sites in UTR](http://www.targetscan.org/cgi-bin/targetscan/vert_60/view_gene.cgi?taxid=9606&rs=NM_022132&members=miR-1246&showcnc=1&shownc=1&showncf=1) |
| [STYX](http://www.ncbi.nlm.nih.gov/sites/entrez?Db=gene&Cmd=ShowDetailView&TermToSearch=6815) | [NM_001130701](http://www.ncbi.nlm.nih.gov/entrez/query.fcgi?cmd=Search&db=nuccore&term==NM_001130701) | serine/threonine/tyrosine interacting protein | [Sites in UTR](http://www.targetscan.org/cgi-bin/targetscan/vert_60/view_gene.cgi?taxid=9606&rs=NM_001130701&members=miR-1246&showcnc=1&shownc=1&showncf=1) |
| [UNC5B](http://www.ncbi.nlm.nih.gov/sites/entrez?Db=gene&Cmd=ShowDetailView&TermToSearch=219699) | [NM_170744](http://www.ncbi.nlm.nih.gov/entrez/query.fcgi?cmd=Search&db=nuccore&term==NM_170744) | unc-5 homolog B (C. elegans) | [Sites in UTR](http://www.targetscan.org/cgi-bin/targetscan/vert_60/view_gene.cgi?taxid=9606&rs=NM_170744&members=miR-1246&showcnc=1&shownc=1&showncf=1) |
| [EFHC2](http://www.ncbi.nlm.nih.gov/sites/entrez?Db=gene&Cmd=ShowDetailView&TermToSearch=80258) | [NM_025184](http://www.ncbi.nlm.nih.gov/entrez/query.fcgi?cmd=Search&db=nuccore&term==NM_025184) | EF-hand domain (C-terminal) containing 2 | [Sites in UTR](http://www.targetscan.org/cgi-bin/targetscan/vert_60/view_gene.cgi?taxid=9606&rs=NM_025184&members=miR-1246&showcnc=1&shownc=1&showncf=1) |
| [YAF2](http://www.ncbi.nlm.nih.gov/sites/entrez?Db=gene&Cmd=ShowDetailView&TermToSearch=10138) | [NM_001190977](http://www.ncbi.nlm.nih.gov/entrez/query.fcgi?cmd=Search&db=nuccore&term==NM_001190977) | YY1 associated factor 2 | [Sites in UTR](http://www.targetscan.org/cgi-bin/targetscan/vert_60/view_gene.cgi?taxid=9606&rs=NM_001190977&members=miR-1246&showcnc=1&shownc=1&showncf=1) |
| [ZNF701](http://www.ncbi.nlm.nih.gov/sites/entrez?Db=gene&Cmd=ShowDetailView&TermToSearch=55762) | [NM_001172655](http://www.ncbi.nlm.nih.gov/entrez/query.fcgi?cmd=Search&db=nuccore&term==NM_001172655) | zinc finger protein 701 | [Sites in UTR](http://www.targetscan.org/cgi-bin/targetscan/vert_60/view_gene.cgi?taxid=9606&rs=NM_001172655&members=miR-1246&showcnc=1&shownc=1&showncf=1) |
| [FAM45A](http://www.ncbi.nlm.nih.gov/sites/entrez?Db=gene&Cmd=ShowDetailView&TermToSearch=404636) | [NM_207009](http://www.ncbi.nlm.nih.gov/entrez/query.fcgi?cmd=Search&db=nuccore&term==NM_207009) | family with sequence similarity 45, member A | [Sites in UTR](http://www.targetscan.org/cgi-bin/targetscan/vert_60/view_gene.cgi?taxid=9606&rs=NM_207009&members=miR-1246&showcnc=1&shownc=1&showncf=1) |
| [GRHL1](http://www.ncbi.nlm.nih.gov/sites/entrez?Db=gene&Cmd=ShowDetailView&TermToSearch=29841) | [NM_198182](http://www.ncbi.nlm.nih.gov/entrez/query.fcgi?cmd=Search&db=nuccore&term==NM_198182) | grainyhead-like 1 (Drosophila) | [Sites in UTR](http://www.targetscan.org/cgi-bin/targetscan/vert_60/view_gene.cgi?taxid=9606&rs=NM_198182&members=miR-1246&showcnc=1&shownc=1&showncf=1) |
| [ZNF816](http://www.ncbi.nlm.nih.gov/sites/entrez?Db=gene&Cmd=ShowDetailView&TermToSearch=125893) | [NM_001031665](http://www.ncbi.nlm.nih.gov/entrez/query.fcgi?cmd=Search&db=nuccore&term==NM_001031665) | zinc finger protein 816 | [Sites in UTR](http://www.targetscan.org/cgi-bin/targetscan/vert_60/view_gene.cgi?taxid=9606&rs=NM_001031665&members=miR-1246&showcnc=1&shownc=1&showncf=1) |
| [MIER3](http://www.ncbi.nlm.nih.gov/sites/entrez?Db=gene&Cmd=ShowDetailView&TermToSearch=166968) | [NM_152622](http://www.ncbi.nlm.nih.gov/entrez/query.fcgi?cmd=Search&db=nuccore&term==NM_152622) | mesoderm induction early response 1, family member 3 | [Sites in UTR](http://www.targetscan.org/cgi-bin/targetscan/vert_60/view_gene.cgi?taxid=9606&rs=NM_152622&members=miR-1246&showcnc=1&shownc=1&showncf=1) |
| [C18orf34](http://www.ncbi.nlm.nih.gov/sites/entrez?Db=gene&Cmd=ShowDetailView&TermToSearch=374864) | [NM_001105528](http://www.ncbi.nlm.nih.gov/entrez/query.fcgi?cmd=Search&db=nuccore&term==NM_001105528) | chromosome 18 open reading frame 34 | [Sites in UTR](http://www.targetscan.org/cgi-bin/targetscan/vert_60/view_gene.cgi?taxid=9606&rs=NM_001105528&members=miR-1246&showcnc=1&shownc=1&showncf=1) |
| [AKAP2](http://www.ncbi.nlm.nih.gov/sites/entrez?Db=gene&Cmd=ShowDetailView&TermToSearch=11217) | [NM_001004065](http://www.ncbi.nlm.nih.gov/entrez/query.fcgi?cmd=Search&db=nuccore&term==NM_001004065) | A kinase (PRKA) anchor protein 2 | [Sites in UTR](http://www.targetscan.org/cgi-bin/targetscan/vert_60/view_gene.cgi?taxid=9606&rs=NM_001004065&members=miR-1246&showcnc=1&shownc=1&showncf=1) |
| [PALM2-AKAP2](http://www.ncbi.nlm.nih.gov/sites/entrez?Db=gene&Cmd=ShowDetailView&TermToSearch=445815) | [NM_007203](http://www.ncbi.nlm.nih.gov/entrez/query.fcgi?cmd=Search&db=nuccore&term==NM_007203) | PALM2-AKAP2 readthrough | [Sites in UTR](http://www.targetscan.org/cgi-bin/targetscan/vert_60/view_gene.cgi?taxid=9606&rs=NM_007203&members=miR-1246&showcnc=1&shownc=1&showncf=1) |
| [SCN3A](http://www.ncbi.nlm.nih.gov/sites/entrez?Db=gene&Cmd=ShowDetailView&TermToSearch=6328) | [NM_001081676](http://www.ncbi.nlm.nih.gov/entrez/query.fcgi?cmd=Search&db=nuccore&term==NM_001081676) | sodium channel, voltage-gated, type III, alpha subunit | [Sites in UTR](http://www.targetscan.org/cgi-bin/targetscan/vert_60/view_gene.cgi?taxid=9606&rs=NM_001081676&members=miR-1246&showcnc=1&shownc=1&showncf=1) |
| [PIK3C2A](http://www.ncbi.nlm.nih.gov/sites/entrez?Db=gene&Cmd=ShowDetailView&TermToSearch=5286) | [NM_002645](http://www.ncbi.nlm.nih.gov/entrez/query.fcgi?cmd=Search&db=nuccore&term==NM_002645) | phosphoinositide-3-kinase, class 2, alpha polypeptide | [Sites in UTR](http://www.targetscan.org/cgi-bin/targetscan/vert_60/view_gene.cgi?taxid=9606&rs=NM_002645&members=miR-1246&showcnc=1&shownc=1&showncf=1) |
| [BEND4](http://www.ncbi.nlm.nih.gov/sites/entrez?Db=gene&Cmd=ShowDetailView&TermToSearch=389206) | [NM_001159547](http://www.ncbi.nlm.nih.gov/entrez/query.fcgi?cmd=Search&db=nuccore&term==NM_001159547) | BEN domain containing 4 | [Sites in UTR](http://www.targetscan.org/cgi-bin/targetscan/vert_60/view_gene.cgi?taxid=9606&rs=NM_001159547&members=miR-1246&showcnc=1&shownc=1&showncf=1) |
| [QTRTD1](http://www.ncbi.nlm.nih.gov/sites/entrez?Db=gene&Cmd=ShowDetailView&TermToSearch=79691) | [NM_024638](http://www.ncbi.nlm.nih.gov/entrez/query.fcgi?cmd=Search&db=nuccore&term==NM_024638) | queuinetRNA-ribosyltransferase domain containing 1 | [Sites in UTR](http://www.targetscan.org/cgi-bin/targetscan/vert_60/view_gene.cgi?taxid=9606&rs=NM_024638&members=miR-1246&showcnc=1&shownc=1&showncf=1) |
| [GEMC1](http://www.ncbi.nlm.nih.gov/sites/entrez?Db=gene&Cmd=ShowDetailView&TermToSearch=647309) | [NM_001146686](http://www.ncbi.nlm.nih.gov/entrez/query.fcgi?cmd=Search&db=nuccore&term==NM_001146686) | geminin coiled-coil domain-containing protein 1 | [Sites in UTR](http://www.targetscan.org/cgi-bin/targetscan/vert_60/view_gene.cgi?taxid=9606&rs=NM_001146686&members=miR-1246&showcnc=1&shownc=1&showncf=1) |
| [DYNC1I1](http://www.ncbi.nlm.nih.gov/sites/entrez?Db=gene&Cmd=ShowDetailView&TermToSearch=1780) | [NM_001135556](http://www.ncbi.nlm.nih.gov/entrez/query.fcgi?cmd=Search&db=nuccore&term==NM_001135556) | dynein, cytoplasmic 1, intermediate chain 1 | [Sites in UTR](http://www.targetscan.org/cgi-bin/targetscan/vert_60/view_gene.cgi?taxid=9606&rs=NM_001135556&members=miR-1246&showcnc=1&shownc=1&showncf=1) |
| [PPP1R11](http://www.ncbi.nlm.nih.gov/sites/entrez?Db=gene&Cmd=ShowDetailView&TermToSearch=6992) | [NM_021959](http://www.ncbi.nlm.nih.gov/entrez/query.fcgi?cmd=Search&db=nuccore&term==NM_021959) | protein phosphatase 1, regulatory (inhibitor) subunit 11 | [Sites in UTR](http://www.targetscan.org/cgi-bin/targetscan/vert_60/view_gene.cgi?taxid=9606&rs=NM_021959&members=miR-1246&showcnc=1&shownc=1&showncf=1) |
| [AXIN2](http://www.ncbi.nlm.nih.gov/sites/entrez?Db=gene&Cmd=ShowDetailView&TermToSearch=8313) | [NM_004655](http://www.ncbi.nlm.nih.gov/entrez/query.fcgi?cmd=Search&db=nuccore&term==NM_004655) | axin 2 | [Sites in UTR](http://www.targetscan.org/cgi-bin/targetscan/vert_60/view_gene.cgi?taxid=9606&rs=NM_004655&members=miR-1246&showcnc=1&shownc=1&showncf=1) |
| [NDFIP1](http://www.ncbi.nlm.nih.gov/sites/entrez?Db=gene&Cmd=ShowDetailView&TermToSearch=80762) | [NM_030571](http://www.ncbi.nlm.nih.gov/entrez/query.fcgi?cmd=Search&db=nuccore&term==NM_030571) | Nedd4 family interacting protein 1 | [Sites in UTR](http://www.targetscan.org/cgi-bin/targetscan/vert_60/view_gene.cgi?taxid=9606&rs=NM_030571&members=miR-1246&showcnc=1&shownc=1&showncf=1) |
| [MC2R](http://www.ncbi.nlm.nih.gov/sites/entrez?Db=gene&Cmd=ShowDetailView&TermToSearch=4158) | [NM_000529](http://www.ncbi.nlm.nih.gov/entrez/query.fcgi?cmd=Search&db=nuccore&term==NM_000529) | melanocortin 2 receptor (adrenocorticotropic hormone) | [Sites in UTR](http://www.targetscan.org/cgi-bin/targetscan/vert_60/view_gene.cgi?taxid=9606&rs=NM_000529&members=miR-1246&showcnc=1&shownc=1&showncf=1) |
| [ZC3H10](http://www.ncbi.nlm.nih.gov/sites/entrez?Db=gene&Cmd=ShowDetailView&TermToSearch=84872) | [NM_032786](http://www.ncbi.nlm.nih.gov/entrez/query.fcgi?cmd=Search&db=nuccore&term==NM_032786) | zinc finger CCCH-type containing 10 | [Sites in UTR](http://www.targetscan.org/cgi-bin/targetscan/vert_60/view_gene.cgi?taxid=9606&rs=NM_032786&members=miR-1246&showcnc=1&shownc=1&showncf=1) |
| [C6orf168](http://www.ncbi.nlm.nih.gov/sites/entrez?Db=gene&Cmd=ShowDetailView&TermToSearch=84553) | [NM_032511](http://www.ncbi.nlm.nih.gov/entrez/query.fcgi?cmd=Search&db=nuccore&term==NM_032511) | chromosome 6 open reading frame 168 | [Sites in UTR](http://www.targetscan.org/cgi-bin/targetscan/vert_60/view_gene.cgi?taxid=9606&rs=NM_032511&members=miR-1246&showcnc=1&shownc=1&showncf=1) |
| [PSD3](http://www.ncbi.nlm.nih.gov/sites/entrez?Db=gene&Cmd=ShowDetailView&TermToSearch=23362) | [NM_015310](http://www.ncbi.nlm.nih.gov/entrez/query.fcgi?cmd=Search&db=nuccore&term==NM_015310) | pleckstrin and Sec7 domain containing 3 | [Sites in UTR](http://www.targetscan.org/cgi-bin/targetscan/vert_60/view_gene.cgi?taxid=9606&rs=NM_015310&members=miR-1246&showcnc=1&shownc=1&showncf=1) |
| [IP6K1](http://www.ncbi.nlm.nih.gov/sites/entrez?Db=gene&Cmd=ShowDetailView&TermToSearch=9807) | [NM_001006115](http://www.ncbi.nlm.nih.gov/entrez/query.fcgi?cmd=Search&db=nuccore&term==NM_001006115) | inositol hexakisphosphate kinase 1 | [Sites in UTR](http://www.targetscan.org/cgi-bin/targetscan/vert_60/view_gene.cgi?taxid=9606&rs=NM_001006115&members=miR-1246&showcnc=1&shownc=1&showncf=1) |
| [AFF3](http://www.ncbi.nlm.nih.gov/sites/entrez?Db=gene&Cmd=ShowDetailView&TermToSearch=3899) | [NM_001025108](http://www.ncbi.nlm.nih.gov/entrez/query.fcgi?cmd=Search&db=nuccore&term==NM_001025108) | AF4/FMR2 family, member 3 | [Sites in UTR](http://www.targetscan.org/cgi-bin/targetscan/vert_60/view_gene.cgi?taxid=9606&rs=NM_001025108&members=miR-1246&showcnc=1&shownc=1&showncf=1) |
| [ENPEP](http://www.ncbi.nlm.nih.gov/sites/entrez?Db=gene&Cmd=ShowDetailView&TermToSearch=2028) | [NM_001977](http://www.ncbi.nlm.nih.gov/entrez/query.fcgi?cmd=Search&db=nuccore&term==NM_001977) | glutamylaminopeptidase (aminopeptidase A) | [Sites in UTR](http://www.targetscan.org/cgi-bin/targetscan/vert_60/view_gene.cgi?taxid=9606&rs=NM_001977&members=miR-1246&showcnc=1&shownc=1&showncf=1) |
| [MAGI2](http://www.ncbi.nlm.nih.gov/sites/entrez?Db=gene&Cmd=ShowDetailView&TermToSearch=9863) | [NM_012301](http://www.ncbi.nlm.nih.gov/entrez/query.fcgi?cmd=Search&db=nuccore&term==NM_012301) | membrane associated guanylate kinase, WW and PDZ domain containing 2 | [Sites in UTR](http://www.targetscan.org/cgi-bin/targetscan/vert_60/view_gene.cgi?taxid=9606&rs=NM_012301&members=miR-1246&showcnc=1&shownc=1&showncf=1) |
| [GRIA1](http://www.ncbi.nlm.nih.gov/sites/entrez?Db=gene&Cmd=ShowDetailView&TermToSearch=2890) | [NM_000827](http://www.ncbi.nlm.nih.gov/entrez/query.fcgi?cmd=Search&db=nuccore&term==NM_000827) | glutamate receptor, ionotropic, AMPA 1 | [Sites in UTR](http://www.targetscan.org/cgi-bin/targetscan/vert_60/view_gene.cgi?taxid=9606&rs=NM_000827&members=miR-1246&showcnc=1&shownc=1&showncf=1) |
| [NIPBL](http://www.ncbi.nlm.nih.gov/sites/entrez?Db=gene&Cmd=ShowDetailView&TermToSearch=25836) | [NM_015384](http://www.ncbi.nlm.nih.gov/entrez/query.fcgi?cmd=Search&db=nuccore&term==NM_015384) | Nipped-B homolog (Drosophila) | [Sites in UTR](http://www.targetscan.org/cgi-bin/targetscan/vert_60/view_gene.cgi?taxid=9606&rs=NM_015384&members=miR-1246&showcnc=1&shownc=1&showncf=1) |
| [ZNF295](http://www.ncbi.nlm.nih.gov/sites/entrez?Db=gene&Cmd=ShowDetailView&TermToSearch=49854) | [NM_001098402](http://www.ncbi.nlm.nih.gov/entrez/query.fcgi?cmd=Search&db=nuccore&term==NM_001098402) | zinc finger protein 295 | [Sites in UTR](http://www.targetscan.org/cgi-bin/targetscan/vert_60/view_gene.cgi?taxid=9606&rs=NM_001098402&members=miR-1246&showcnc=1&shownc=1&showncf=1) |
| [GSK3B](http://www.ncbi.nlm.nih.gov/sites/entrez?Db=gene&Cmd=ShowDetailView&TermToSearch=2932) | [NM_001146156](http://www.ncbi.nlm.nih.gov/entrez/query.fcgi?cmd=Search&db=nuccore&term==NM_001146156) | glycogen synthase kinase 3 beta | [Sites in UTR](http://www.targetscan.org/cgi-bin/targetscan/vert_60/view_gene.cgi?taxid=9606&rs=NM_001146156&members=miR-1246&showcnc=1&shownc=1&showncf=1) |
| [DYRK1A](http://www.ncbi.nlm.nih.gov/sites/entrez?Db=gene&Cmd=ShowDetailView&TermToSearch=1859) | [NM_001396](http://www.ncbi.nlm.nih.gov/entrez/query.fcgi?cmd=Search&db=nuccore&term==NM_001396) | dual-specificity tyrosine-(Y)-phosphorylation regulated kinase 1A | [Sites in UTR](http://www.targetscan.org/cgi-bin/targetscan/vert_60/view_gene.cgi?taxid=9606&rs=NM_001396&members=miR-1246&showcnc=1&shownc=1&showncf=1) |
| [CCNG2](http://www.ncbi.nlm.nih.gov/sites/entrez?Db=gene&Cmd=ShowDetailView&TermToSearch=901) | [NM_004354](http://www.ncbi.nlm.nih.gov/entrez/query.fcgi?cmd=Search&db=nuccore&term==NM_004354) | cyclin G2 | [Sites in UTR](http://www.targetscan.org/cgi-bin/targetscan/vert_60/view_gene.cgi?taxid=9606&rs=NM_004354&members=miR-1246&showcnc=1&shownc=1&showncf=1) |
| [EIF4E](http://www.ncbi.nlm.nih.gov/sites/entrez?Db=gene&Cmd=ShowDetailView&TermToSearch=1977) | [NM_001130678](http://www.ncbi.nlm.nih.gov/entrez/query.fcgi?cmd=Search&db=nuccore&term==NM_001130678) | eukaryotic translation initiation factor 4E | [Sites in UTR](http://www.targetscan.org/cgi-bin/targetscan/vert_60/view_gene.cgi?taxid=9606&rs=NM_001130678&members=miR-1246&showcnc=1&shownc=1&showncf=1) |
| [CDH2](http://www.ncbi.nlm.nih.gov/sites/entrez?Db=gene&Cmd=ShowDetailView&TermToSearch=1000) | [NM_001792](http://www.ncbi.nlm.nih.gov/entrez/query.fcgi?cmd=Search&db=nuccore&term==NM_001792) | cadherin 2, type 1, N-cadherin (neuronal) | [Sites in UTR](http://www.targetscan.org/cgi-bin/targetscan/vert_60/view_gene.cgi?taxid=9606&rs=NM_001792&members=miR-1246&showcnc=1&shownc=1&showncf=1) |
| [SLC43A2](http://www.ncbi.nlm.nih.gov/sites/entrez?Db=gene&Cmd=ShowDetailView&TermToSearch=124935) | [NM_152346](http://www.ncbi.nlm.nih.gov/entrez/query.fcgi?cmd=Search&db=nuccore&term==NM_152346) | solute carrier family 43, member 2 | [Sites in UTR](http://www.targetscan.org/cgi-bin/targetscan/vert_60/view_gene.cgi?taxid=9606&rs=NM_152346&members=miR-1246&showcnc=1&shownc=1&showncf=1) |
| [SLC12A2](http://www.ncbi.nlm.nih.gov/sites/entrez?Db=gene&Cmd=ShowDetailView&TermToSearch=6558) | [NM_001046](http://www.ncbi.nlm.nih.gov/entrez/query.fcgi?cmd=Search&db=nuccore&term==NM_001046) | solute carrier family 12 (sodium/potassium/chloride transporters), member 2 | [Sites in UTR](http://www.targetscan.org/cgi-bin/targetscan/vert_60/view_gene.cgi?taxid=9606&rs=NM_001046&members=miR-1246&showcnc=1&shownc=1&showncf=1) |
| [C12orf12](http://www.ncbi.nlm.nih.gov/sites/entrez?Db=gene&Cmd=ShowDetailView&TermToSearch=196477) | [NM_152638](http://www.ncbi.nlm.nih.gov/entrez/query.fcgi?cmd=Search&db=nuccore&term==NM_152638) | chromosome 12 open reading frame 12 | [Sites in UTR](http://www.targetscan.org/cgi-bin/targetscan/vert_60/view_gene.cgi?taxid=9606&rs=NM_152638&members=miR-1246&showcnc=1&shownc=1&showncf=1) |
| [OLFML2B](http://www.ncbi.nlm.nih.gov/sites/entrez?Db=gene&Cmd=ShowDetailView&TermToSearch=25903) | [NM_015441](http://www.ncbi.nlm.nih.gov/entrez/query.fcgi?cmd=Search&db=nuccore&term==NM_015441) | olfactomedin-like 2B | [Sites in UTR](http://www.targetscan.org/cgi-bin/targetscan/vert_60/view_gene.cgi?taxid=9606&rs=NM_015441&members=miR-1246&showcnc=1&shownc=1&showncf=1) |
| [CBX5](http://www.ncbi.nlm.nih.gov/sites/entrez?Db=gene&Cmd=ShowDetailView&TermToSearch=23468) | [NM_001127321](http://www.ncbi.nlm.nih.gov/entrez/query.fcgi?cmd=Search&db=nuccore&term==NM_001127321) | chromobox homolog 5 | [Sites in UTR](http://www.targetscan.org/cgi-bin/targetscan/vert_60/view_gene.cgi?taxid=9606&rs=NM_001127321&members=miR-1246&showcnc=1&shownc=1&showncf=1) |
| [PCBP2](http://www.ncbi.nlm.nih.gov/sites/entrez?Db=gene&Cmd=ShowDetailView&TermToSearch=5094) | [NM_001098620](http://www.ncbi.nlm.nih.gov/entrez/query.fcgi?cmd=Search&db=nuccore&term==NM_001098620) | poly(rC) binding protein 2 | [Sites in UTR](http://www.targetscan.org/cgi-bin/targetscan/vert_60/view_gene.cgi?taxid=9606&rs=NM_001098620&members=miR-1246&showcnc=1&shownc=1&showncf=1) |
| [WASF3](http://www.ncbi.nlm.nih.gov/sites/entrez?Db=gene&Cmd=ShowDetailView&TermToSearch=10810) | [NM_006646](http://www.ncbi.nlm.nih.gov/entrez/query.fcgi?cmd=Search&db=nuccore&term==NM_006646) | WAS protein family, member 3 | [Sites in UTR](http://www.targetscan.org/cgi-bin/targetscan/vert_60/view_gene.cgi?taxid=9606&rs=NM_006646&members=miR-1246&showcnc=1&shownc=1&showncf=1) |
| [ZCRB1](http://www.ncbi.nlm.nih.gov/sites/entrez?Db=gene&Cmd=ShowDetailView&TermToSearch=85437) | [NM_033114](http://www.ncbi.nlm.nih.gov/entrez/query.fcgi?cmd=Search&db=nuccore&term==NM_033114) | zinc finger CCHC-type and RNA binding motif 1 | [Sites in UTR](http://www.targetscan.org/cgi-bin/targetscan/vert_60/view_gene.cgi?taxid=9606&rs=NM_033114&members=miR-1246&showcnc=1&shownc=1&showncf=1) |
| [DHX33](http://www.ncbi.nlm.nih.gov/sites/entrez?Db=gene&Cmd=ShowDetailView&TermToSearch=56919) | [NM_001199699](http://www.ncbi.nlm.nih.gov/entrez/query.fcgi?cmd=Search&db=nuccore&term==NM_001199699) | DEAH (Asp-Glu-Ala-His) box polypeptide 33 | [Sites in UTR](http://www.targetscan.org/cgi-bin/targetscan/vert_60/view_gene.cgi?taxid=9606&rs=NM_001199699&members=miR-1246&showcnc=1&shownc=1&showncf=1) |
| [AQP1](http://www.ncbi.nlm.nih.gov/sites/entrez?Db=gene&Cmd=ShowDetailView&TermToSearch=358) | [NM_001185060](http://www.ncbi.nlm.nih.gov/entrez/query.fcgi?cmd=Search&db=nuccore&term==NM_001185060) | aquaporin 1 (Colton blood group) | [Sites in UTR](http://www.targetscan.org/cgi-bin/targetscan/vert_60/view_gene.cgi?taxid=9606&rs=NM_001185060&members=miR-1246&showcnc=1&shownc=1&showncf=1) |
| [CT62](http://www.ncbi.nlm.nih.gov/sites/entrez?Db=gene&Cmd=ShowDetailView&TermToSearch=196993) | [NM_001102658](http://www.ncbi.nlm.nih.gov/entrez/query.fcgi?cmd=Search&db=nuccore&term==NM_001102658) | cancer/testis antigen 62 | [Sites in UTR](http://www.targetscan.org/cgi-bin/targetscan/vert_60/view_gene.cgi?taxid=9606&rs=NM_001102658&members=miR-1246&showcnc=1&shownc=1&showncf=1) |
| [GNRHR](http://www.ncbi.nlm.nih.gov/sites/entrez?Db=gene&Cmd=ShowDetailView&TermToSearch=2798) | [NM_000406](http://www.ncbi.nlm.nih.gov/entrez/query.fcgi?cmd=Search&db=nuccore&term==NM_000406) | gonadotropin-releasing hormone receptor | [Sites in UTR](http://www.targetscan.org/cgi-bin/targetscan/vert_60/view_gene.cgi?taxid=9606&rs=NM_000406&members=miR-1246&showcnc=1&shownc=1&showncf=1) |
| [TMEM33](http://www.ncbi.nlm.nih.gov/sites/entrez?Db=gene&Cmd=ShowDetailView&TermToSearch=55161) | [NM_018126](http://www.ncbi.nlm.nih.gov/entrez/query.fcgi?cmd=Search&db=nuccore&term==NM_018126) | transmembrane protein 33 | [Sites in UTR](http://www.targetscan.org/cgi-bin/targetscan/vert_60/view_gene.cgi?taxid=9606&rs=NM_018126&members=miR-1246&showcnc=1&shownc=1&showncf=1) |
| [ATXN7L3](http://www.ncbi.nlm.nih.gov/sites/entrez?Db=gene&Cmd=ShowDetailView&TermToSearch=56970) | [NM_001098833](http://www.ncbi.nlm.nih.gov/entrez/query.fcgi?cmd=Search&db=nuccore&term==NM_001098833) | ataxin 7-like 3 | [Sites in UTR](http://www.targetscan.org/cgi-bin/targetscan/vert_60/view_gene.cgi?taxid=9606&rs=NM_001098833&members=miR-1246&showcnc=1&shownc=1&showncf=1) |
| [CALM2](http://www.ncbi.nlm.nih.gov/sites/entrez?Db=gene&Cmd=ShowDetailView&TermToSearch=805) | [NM_001743](http://www.ncbi.nlm.nih.gov/entrez/query.fcgi?cmd=Search&db=nuccore&term==NM_001743) | calmodulin 2 (phosphorylase kinase, delta) | [Sites in UTR](http://www.targetscan.org/cgi-bin/targetscan/vert_60/view_gene.cgi?taxid=9606&rs=NM_001743&members=miR-1246&showcnc=1&shownc=1&showncf=1) |
| [ZFP36L1](http://www.ncbi.nlm.nih.gov/sites/entrez?Db=gene&Cmd=ShowDetailView&TermToSearch=677) | [NM_004926](http://www.ncbi.nlm.nih.gov/entrez/query.fcgi?cmd=Search&db=nuccore&term==NM_004926) | zinc finger protein 36, C3H type-like 1 | [Sites in UTR](http://www.targetscan.org/cgi-bin/targetscan/vert_60/view_gene.cgi?taxid=9606&rs=NM_004926&members=miR-1246&showcnc=1&shownc=1&showncf=1) |
| [NHLH2](http://www.ncbi.nlm.nih.gov/sites/entrez?Db=gene&Cmd=ShowDetailView&TermToSearch=4808) | [NM_001111061](http://www.ncbi.nlm.nih.gov/entrez/query.fcgi?cmd=Search&db=nuccore&term==NM_001111061) | nescient helix loop helix 2 | [Sites in UTR](http://www.targetscan.org/cgi-bin/targetscan/vert_60/view_gene.cgi?taxid=9606&rs=NM_001111061&members=miR-1246&showcnc=1&shownc=1&showncf=1) |
| [KCNAB1](http://www.ncbi.nlm.nih.gov/sites/entrez?Db=gene&Cmd=ShowDetailView&TermToSearch=7881) | [NM_003471](http://www.ncbi.nlm.nih.gov/entrez/query.fcgi?cmd=Search&db=nuccore&term==NM_003471) | potassium voltage-gated channel, shaker-related subfamily, beta member 1 | [Sites in UTR](http://www.targetscan.org/cgi-bin/targetscan/vert_60/view_gene.cgi?taxid=9606&rs=NM_003471&members=miR-1246&showcnc=1&shownc=1&showncf=1) |
| [BAHD1](http://www.ncbi.nlm.nih.gov/sites/entrez?Db=gene&Cmd=ShowDetailView&TermToSearch=22893) | [NM_014952](http://www.ncbi.nlm.nih.gov/entrez/query.fcgi?cmd=Search&db=nuccore&term==NM_014952) | bromo adjacent homology domain containing 1 | [Sites in UTR](http://www.targetscan.org/cgi-bin/targetscan/vert_60/view_gene.cgi?taxid=9606&rs=NM_014952&members=miR-1246&showcnc=1&shownc=1&showncf=1) |
| [GTF2I](http://www.ncbi.nlm.nih.gov/sites/entrez?Db=gene&Cmd=ShowDetailView&TermToSearch=2969) | [NM_001163636](http://www.ncbi.nlm.nih.gov/entrez/query.fcgi?cmd=Search&db=nuccore&term==NM_001163636) | general transcription factor IIi | [Sites in UTR](http://www.targetscan.org/cgi-bin/targetscan/vert_60/view_gene.cgi?taxid=9606&rs=NM_001163636&members=miR-1246&showcnc=1&shownc=1&showncf=1) |
| [ANKFY1](http://www.ncbi.nlm.nih.gov/sites/entrez?Db=gene&Cmd=ShowDetailView&TermToSearch=51479) | [NM_016376](http://www.ncbi.nlm.nih.gov/entrez/query.fcgi?cmd=Search&db=nuccore&term==NM_016376) | ankyrin repeat and FYVE domain containing 1 | [Sites in UTR](http://www.targetscan.org/cgi-bin/targetscan/vert_60/view_gene.cgi?taxid=9606&rs=NM_016376&members=miR-1246&showcnc=1&shownc=1&showncf=1) |
| [DNAJC3](http://www.ncbi.nlm.nih.gov/sites/entrez?Db=gene&Cmd=ShowDetailView&TermToSearch=5611) | [NM_006260](http://www.ncbi.nlm.nih.gov/entrez/query.fcgi?cmd=Search&db=nuccore&term==NM_006260) | DnaJ (Hsp40) homolog, subfamily C, member 3 | [Sites in UTR](http://www.targetscan.org/cgi-bin/targetscan/vert_60/view_gene.cgi?taxid=9606&rs=NM_006260&members=miR-1246&showcnc=1&shownc=1&showncf=1) |
| [NRP2](http://www.ncbi.nlm.nih.gov/sites/entrez?Db=gene&Cmd=ShowDetailView&TermToSearch=8828) | [NM_003872](http://www.ncbi.nlm.nih.gov/entrez/query.fcgi?cmd=Search&db=nuccore&term==NM_003872) | neuropilin 2 | [Sites in UTR](http://www.targetscan.org/cgi-bin/targetscan/vert_60/view_gene.cgi?taxid=9606&rs=NM_003872&members=miR-1246&showcnc=1&shownc=1&showncf=1) |
| [CALB1](http://www.ncbi.nlm.nih.gov/sites/entrez?Db=gene&Cmd=ShowDetailView&TermToSearch=793) | [NM_004929](http://www.ncbi.nlm.nih.gov/entrez/query.fcgi?cmd=Search&db=nuccore&term==NM_004929) | calbindin 1, 28kDa | [Sites in UTR](http://www.targetscan.org/cgi-bin/targetscan/vert_60/view_gene.cgi?taxid=9606&rs=NM_004929&members=miR-1246&showcnc=1&shownc=1&showncf=1) |
| [NUP153](http://www.ncbi.nlm.nih.gov/sites/entrez?Db=gene&Cmd=ShowDetailView&TermToSearch=9972) | [NM_005124](http://www.ncbi.nlm.nih.gov/entrez/query.fcgi?cmd=Search&db=nuccore&term==NM_005124) | nucleoporin 153kDa | [Sites in UTR](http://www.targetscan.org/cgi-bin/targetscan/vert_60/view_gene.cgi?taxid=9606&rs=NM_005124&members=miR-1246&showcnc=1&shownc=1&showncf=1) |
| [H2AFJ](http://www.ncbi.nlm.nih.gov/sites/entrez?Db=gene&Cmd=ShowDetailView&TermToSearch=55766) | [NM_177925](http://www.ncbi.nlm.nih.gov/entrez/query.fcgi?cmd=Search&db=nuccore&term==NM_177925) | H2A histone family, member J | [Sites in UTR](http://www.targetscan.org/cgi-bin/targetscan/vert_60/view_gene.cgi?taxid=9606&rs=NM_177925&members=miR-1246&showcnc=1&shownc=1&showncf=1) |
| [ATP2B1](http://www.ncbi.nlm.nih.gov/sites/entrez?Db=gene&Cmd=ShowDetailView&TermToSearch=490) | [NM_001001323](http://www.ncbi.nlm.nih.gov/entrez/query.fcgi?cmd=Search&db=nuccore&term==NM_001001323) | ATPase, Ca++ transporting, plasma membrane 1 | [Sites in UTR](http://www.targetscan.org/cgi-bin/targetscan/vert_60/view_gene.cgi?taxid=9606&rs=NM_001001323&members=miR-1246&showcnc=1&shownc=1&showncf=1) |
| [TMEM132D](http://www.ncbi.nlm.nih.gov/sites/entrez?Db=gene&Cmd=ShowDetailView&TermToSearch=121256) | [NM_133448](http://www.ncbi.nlm.nih.gov/entrez/query.fcgi?cmd=Search&db=nuccore&term==NM_133448) | transmembrane protein 132D | [Sites in UTR](http://www.targetscan.org/cgi-bin/targetscan/vert_60/view_gene.cgi?taxid=9606&rs=NM_133448&members=miR-1246&showcnc=1&shownc=1&showncf=1) |
| [KIAA0240](http://www.ncbi.nlm.nih.gov/sites/entrez?Db=gene&Cmd=ShowDetailView&TermToSearch=23506) | [NM_015349](http://www.ncbi.nlm.nih.gov/entrez/query.fcgi?cmd=Search&db=nuccore&term==NM_015349) | KIAA0240 | [Sites in UTR](http://www.targetscan.org/cgi-bin/targetscan/vert_60/view_gene.cgi?taxid=9606&rs=NM_015349&members=miR-1246&showcnc=1&shownc=1&showncf=1) |
| [ZNF770](http://www.ncbi.nlm.nih.gov/sites/entrez?Db=gene&Cmd=ShowDetailView&TermToSearch=54989) | [NM_014106](http://www.ncbi.nlm.nih.gov/entrez/query.fcgi?cmd=Search&db=nuccore&term==NM_014106) | zinc finger protein 770 | [Sites in UTR](http://www.targetscan.org/cgi-bin/targetscan/vert_60/view_gene.cgi?taxid=9606&rs=NM_014106&members=miR-1246&showcnc=1&shownc=1&showncf=1) |
| [SPTY2D1](http://www.ncbi.nlm.nih.gov/sites/entrez?Db=gene&Cmd=ShowDetailView&TermToSearch=144108) | [NM_194285](http://www.ncbi.nlm.nih.gov/entrez/query.fcgi?cmd=Search&db=nuccore&term==NM_194285) | SPT2, Suppressor of Ty, domain containing 1 (S. cerevisiae) | [Sites in UTR](http://www.targetscan.org/cgi-bin/targetscan/vert_60/view_gene.cgi?taxid=9606&rs=NM_194285&members=miR-1246&showcnc=1&shownc=1&showncf=1) |
| [EDA](http://www.ncbi.nlm.nih.gov/sites/entrez?Db=gene&Cmd=ShowDetailView&TermToSearch=1896) | [NM_001005609](http://www.ncbi.nlm.nih.gov/entrez/query.fcgi?cmd=Search&db=nuccore&term==NM_001005609) | ectodysplasin A | [Sites in UTR](http://www.targetscan.org/cgi-bin/targetscan/vert_60/view_gene.cgi?taxid=9606&rs=NM_001005609&members=miR-1246&showcnc=1&shownc=1&showncf=1) |
| [SLC17A8](http://www.ncbi.nlm.nih.gov/sites/entrez?Db=gene&Cmd=ShowDetailView&TermToSearch=246213) | [NM_001145288](http://www.ncbi.nlm.nih.gov/entrez/query.fcgi?cmd=Search&db=nuccore&term==NM_001145288) | solute carrier family 17 (sodium-dependent inorganic phosphate cotransporter), member 8 | [Sites in UTR](http://www.targetscan.org/cgi-bin/targetscan/vert_60/view_gene.cgi?taxid=9606&rs=NM_001145288&members=miR-1246&showcnc=1&shownc=1&showncf=1) |
| [PCDHB5](http://www.ncbi.nlm.nih.gov/sites/entrez?Db=gene&Cmd=ShowDetailView&TermToSearch=26167) | [NM_015669](http://www.ncbi.nlm.nih.gov/entrez/query.fcgi?cmd=Search&db=nuccore&term==NM_015669) | protocadherin beta 5 | [Sites in UTR](http://www.targetscan.org/cgi-bin/targetscan/vert_60/view_gene.cgi?taxid=9606&rs=NM_015669&members=miR-1246&showcnc=1&shownc=1&showncf=1) |
| [EIF2AK3](http://www.ncbi.nlm.nih.gov/sites/entrez?Db=gene&Cmd=ShowDetailView&TermToSearch=9451) | [NM_004836](http://www.ncbi.nlm.nih.gov/entrez/query.fcgi?cmd=Search&db=nuccore&term==NM_004836) | eukaryotic translation initiation factor 2-alpha kinase 3 | [Sites in UTR](http://www.targetscan.org/cgi-bin/targetscan/vert_60/view_gene.cgi?taxid=9606&rs=NM_004836&members=miR-1246&showcnc=1&shownc=1&showncf=1) |
| [SLC38A2](http://www.ncbi.nlm.nih.gov/sites/entrez?Db=gene&Cmd=ShowDetailView&TermToSearch=54407) | [NM_018976](http://www.ncbi.nlm.nih.gov/entrez/query.fcgi?cmd=Search&db=nuccore&term==NM_018976) | solute carrier family 38, member 2 | [Sites in UTR](http://www.targetscan.org/cgi-bin/targetscan/vert_60/view_gene.cgi?taxid=9606&rs=NM_018976&members=miR-1246&showcnc=1&shownc=1&showncf=1) |
| [SH2B3](http://www.ncbi.nlm.nih.gov/sites/entrez?Db=gene&Cmd=ShowDetailView&TermToSearch=10019) | [NM_005475](http://www.ncbi.nlm.nih.gov/entrez/query.fcgi?cmd=Search&db=nuccore&term==NM_005475) | SH2B adaptor protein 3 | [Sites in UTR](http://www.targetscan.org/cgi-bin/targetscan/vert_60/view_gene.cgi?taxid=9606&rs=NM_005475&members=miR-1246&showcnc=1&shownc=1&showncf=1) |
| [TAF9B](http://www.ncbi.nlm.nih.gov/sites/entrez?Db=gene&Cmd=ShowDetailView&TermToSearch=51616) | [NM_015975](http://www.ncbi.nlm.nih.gov/entrez/query.fcgi?cmd=Search&db=nuccore&term==NM_015975) | TAF9B RNA polymerase II, TATA box binding protein (TBP)-associated factor, 31kDa | [Sites in UTR](http://www.targetscan.org/cgi-bin/targetscan/vert_60/view_gene.cgi?taxid=9606&rs=NM_015975&members=miR-1246&showcnc=1&shownc=1&showncf=1) |
| [HOMEZ](http://www.ncbi.nlm.nih.gov/sites/entrez?Db=gene&Cmd=ShowDetailView&TermToSearch=57594) | [NM_020834](http://www.ncbi.nlm.nih.gov/entrez/query.fcgi?cmd=Search&db=nuccore&term==NM_020834) | homeobox and leucine zipper encoding | [Sites in UTR](http://www.targetscan.org/cgi-bin/targetscan/vert_60/view_gene.cgi?taxid=9606&rs=NM_020834&members=miR-1246&showcnc=1&shownc=1&showncf=1) |
| [PTCH1](http://www.ncbi.nlm.nih.gov/sites/entrez?Db=gene&Cmd=ShowDetailView&TermToSearch=5727) | [NM_000264](http://www.ncbi.nlm.nih.gov/entrez/query.fcgi?cmd=Search&db=nuccore&term==NM_000264) | patched 1 | [Sites in UTR](http://www.targetscan.org/cgi-bin/targetscan/vert_60/view_gene.cgi?taxid=9606&rs=NM_000264&members=miR-1246&showcnc=1&shownc=1&showncf=1) |
| [KDM5A](http://www.ncbi.nlm.nih.gov/sites/entrez?Db=gene&Cmd=ShowDetailView&TermToSearch=5927) | [NM_001042603](http://www.ncbi.nlm.nih.gov/entrez/query.fcgi?cmd=Search&db=nuccore&term==NM_001042603) | lysine (K)-specific demethylase 5A | [Sites in UTR](http://www.targetscan.org/cgi-bin/targetscan/vert_60/view_gene.cgi?taxid=9606&rs=NM_001042603&members=miR-1246&showcnc=1&shownc=1&showncf=1) |
| [SEMA6A](http://www.ncbi.nlm.nih.gov/sites/entrez?Db=gene&Cmd=ShowDetailView&TermToSearch=57556) | [NM_020796](http://www.ncbi.nlm.nih.gov/entrez/query.fcgi?cmd=Search&db=nuccore&term==NM_020796) | sema domain, transmembrane domain (TM), and cytoplasmic domain, (semaphorin) 6A | [Sites in UTR](http://www.targetscan.org/cgi-bin/targetscan/vert_60/view_gene.cgi?taxid=9606&rs=NM_020796&members=miR-1246&showcnc=1&shownc=1&showncf=1) |
| [STAG3L4](http://www.ncbi.nlm.nih.gov/sites/entrez?Db=gene&Cmd=ShowDetailView&TermToSearch=64940) | [NM_022906](http://www.ncbi.nlm.nih.gov/entrez/query.fcgi?cmd=Search&db=nuccore&term==NM_022906) | stromal antigen 3-like 4 | [Sites in UTR](http://www.targetscan.org/cgi-bin/targetscan/vert_60/view_gene.cgi?taxid=9606&rs=NM_022906&members=miR-1246&showcnc=1&shownc=1&showncf=1) |
| [NUP50](http://www.ncbi.nlm.nih.gov/sites/entrez?Db=gene&Cmd=ShowDetailView&TermToSearch=10762) | [NM_007172](http://www.ncbi.nlm.nih.gov/entrez/query.fcgi?cmd=Search&db=nuccore&term==NM_007172) | nucleoporin 50kDa | [Sites in UTR](http://www.targetscan.org/cgi-bin/targetscan/vert_60/view_gene.cgi?taxid=9606&rs=NM_007172&members=miR-1246&showcnc=1&shownc=1&showncf=1) |
| [SHISA9](http://www.ncbi.nlm.nih.gov/sites/entrez?Db=gene&Cmd=ShowDetailView&TermToSearch=729993) | [NM_001145204](http://www.ncbi.nlm.nih.gov/entrez/query.fcgi?cmd=Search&db=nuccore&term==NM_001145204) | shisa homolog 9 (Xenopuslaevis) | [Sites in UTR](http://www.targetscan.org/cgi-bin/targetscan/vert_60/view_gene.cgi?taxid=9606&rs=NM_001145204&members=miR-1246&showcnc=1&shownc=1&showncf=1) |
| [TEAD1](http://www.ncbi.nlm.nih.gov/sites/entrez?Db=gene&Cmd=ShowDetailView&TermToSearch=7003) | [NM_021961](http://www.ncbi.nlm.nih.gov/entrez/query.fcgi?cmd=Search&db=nuccore&term==NM_021961) | TEA domain family member 1 (SV40 transcriptional enhancer factor) | [Sites in UTR](http://www.targetscan.org/cgi-bin/targetscan/vert_60/view_gene.cgi?taxid=9606&rs=NM_021961&members=miR-1246&showcnc=1&shownc=1&showncf=1) |
| [C6orf35](http://www.ncbi.nlm.nih.gov/sites/entrez?Db=gene&Cmd=ShowDetailView&TermToSearch=729515) | [NM_018452](http://www.ncbi.nlm.nih.gov/entrez/query.fcgi?cmd=Search&db=nuccore&term==NM_018452) | chromosome 6 open reading frame 35 | [Sites in UTR](http://www.targetscan.org/cgi-bin/targetscan/vert_60/view_gene.cgi?taxid=9606&rs=NM_018452&members=miR-1246&showcnc=1&shownc=1&showncf=1) |
| [LRRTM2](http://www.ncbi.nlm.nih.gov/sites/entrez?Db=gene&Cmd=ShowDetailView&TermToSearch=26045) | [NM_015564](http://www.ncbi.nlm.nih.gov/entrez/query.fcgi?cmd=Search&db=nuccore&term==NM_015564) | leucine rich repeat transmembrane neuronal 2 | [Sites in UTR](http://www.targetscan.org/cgi-bin/targetscan/vert_60/view_gene.cgi?taxid=9606&rs=NM_015564&members=miR-1246&showcnc=1&shownc=1&showncf=1) |
| [SIAH3](http://www.ncbi.nlm.nih.gov/sites/entrez?Db=gene&Cmd=ShowDetailView&TermToSearch=283514) | [NM_198849](http://www.ncbi.nlm.nih.gov/entrez/query.fcgi?cmd=Search&db=nuccore&term==NM_198849) | seven in absentia homolog 3 (Drosophila) | [Sites in UTR](http://www.targetscan.org/cgi-bin/targetscan/vert_60/view_gene.cgi?taxid=9606&rs=NM_198849&members=miR-1246&showcnc=1&shownc=1&showncf=1) |
| [MEX3C](http://www.ncbi.nlm.nih.gov/sites/entrez?Db=gene&Cmd=ShowDetailView&TermToSearch=51320) | [NM_016626](http://www.ncbi.nlm.nih.gov/entrez/query.fcgi?cmd=Search&db=nuccore&term==NM_016626) | mex-3 homolog C (C. elegans) | [Sites in UTR](http://www.targetscan.org/cgi-bin/targetscan/vert_60/view_gene.cgi?taxid=9606&rs=NM_016626&members=miR-1246&showcnc=1&shownc=1&showncf=1) |
| [HIPK2](http://www.ncbi.nlm.nih.gov/sites/entrez?Db=gene&Cmd=ShowDetailView&TermToSearch=28996) | [NM_001113239](http://www.ncbi.nlm.nih.gov/entrez/query.fcgi?cmd=Search&db=nuccore&term==NM_001113239) | homeodomain interacting protein kinase 2 | [Sites in UTR](http://www.targetscan.org/cgi-bin/targetscan/vert_60/view_gene.cgi?taxid=9606&rs=NM_001113239&members=miR-1246&showcnc=1&shownc=1&showncf=1) |
| [FMN1](http://www.ncbi.nlm.nih.gov/sites/entrez?Db=gene&Cmd=ShowDetailView&TermToSearch=342184) | [NM_001103184](http://www.ncbi.nlm.nih.gov/entrez/query.fcgi?cmd=Search&db=nuccore&term==NM_001103184) | formin 1 | [Sites in UTR](http://www.targetscan.org/cgi-bin/targetscan/vert_60/view_gene.cgi?taxid=9606&rs=NM_001103184&members=miR-1246&showcnc=1&shownc=1&showncf=1) |
| [ADAMTS6](http://www.ncbi.nlm.nih.gov/sites/entrez?Db=gene&Cmd=ShowDetailView&TermToSearch=11174) | [NM_197941](http://www.ncbi.nlm.nih.gov/entrez/query.fcgi?cmd=Search&db=nuccore&term==NM_197941) | ADAM metallopeptidase with thrombospondin type 1 motif, 6 | [Sites in UTR](http://www.targetscan.org/cgi-bin/targetscan/vert_60/view_gene.cgi?taxid=9606&rs=NM_197941&members=miR-1246&showcnc=1&shownc=1&showncf=1) |
| [TMTC3](http://www.ncbi.nlm.nih.gov/sites/entrez?Db=gene&Cmd=ShowDetailView&TermToSearch=160418) | [NM_181783](http://www.ncbi.nlm.nih.gov/entrez/query.fcgi?cmd=Search&db=nuccore&term==NM_181783) | transmembrane and tetratricopeptide repeat containing 3 | [Sites in UTR](http://www.targetscan.org/cgi-bin/targetscan/vert_60/view_gene.cgi?taxid=9606&rs=NM_181783&members=miR-1246&showcnc=1&shownc=1&showncf=1) |
| [ADD2](http://www.ncbi.nlm.nih.gov/sites/entrez?Db=gene&Cmd=ShowDetailView&TermToSearch=119) | [NM_001185054](http://www.ncbi.nlm.nih.gov/entrez/query.fcgi?cmd=Search&db=nuccore&term==NM_001185054) | adducin 2 (beta) | [Sites in UTR](http://www.targetscan.org/cgi-bin/targetscan/vert_60/view_gene.cgi?taxid=9606&rs=NM_001185054&members=miR-1246&showcnc=1&shownc=1&showncf=1) |
| [CLLU1](http://www.ncbi.nlm.nih.gov/sites/entrez?Db=gene&Cmd=ShowDetailView&TermToSearch=574028) | [NM_001025233](http://www.ncbi.nlm.nih.gov/entrez/query.fcgi?cmd=Search&db=nuccore&term==NM_001025233) | chronic lymphocytic leukemia up-regulated 1 | [Sites in UTR](http://www.targetscan.org/cgi-bin/targetscan/vert_60/view_gene.cgi?taxid=9606&rs=NM_001025233&members=miR-1246&showcnc=1&shownc=1&showncf=1) |
| [ARHGEF26](http://www.ncbi.nlm.nih.gov/sites/entrez?Db=gene&Cmd=ShowDetailView&TermToSearch=26084) | [NM_015595](http://www.ncbi.nlm.nih.gov/entrez/query.fcgi?cmd=Search&db=nuccore&term==NM_015595) | Rho guanine nucleotide exchange factor (GEF) 26 | [Sites in UTR](http://www.targetscan.org/cgi-bin/targetscan/vert_60/view_gene.cgi?taxid=9606&rs=NM_015595&members=miR-1246&showcnc=1&shownc=1&showncf=1) |
| [SYT11](http://www.ncbi.nlm.nih.gov/sites/entrez?Db=gene&Cmd=ShowDetailView&TermToSearch=23208) | [NM_152280](http://www.ncbi.nlm.nih.gov/entrez/query.fcgi?cmd=Search&db=nuccore&term==NM_152280) | synaptotagmin XI | [Sites in UTR](http://www.targetscan.org/cgi-bin/targetscan/vert_60/view_gene.cgi?taxid=9606&rs=NM_152280&members=miR-1246&showcnc=1&shownc=1&showncf=1) |
| [CXorf36](http://www.ncbi.nlm.nih.gov/sites/entrez?Db=gene&Cmd=ShowDetailView&TermToSearch=79742) | [NM_024689](http://www.ncbi.nlm.nih.gov/entrez/query.fcgi?cmd=Search&db=nuccore&term==NM_024689) | chromosome X open reading frame 36 | [Sites in UTR](http://www.targetscan.org/cgi-bin/targetscan/vert_60/view_gene.cgi?taxid=9606&rs=NM_024689&members=miR-1246&showcnc=1&shownc=1&showncf=1) |
| [METAP2](http://www.ncbi.nlm.nih.gov/sites/entrez?Db=gene&Cmd=ShowDetailView&TermToSearch=10988) | [NM_006838](http://www.ncbi.nlm.nih.gov/entrez/query.fcgi?cmd=Search&db=nuccore&term==NM_006838) | methionylaminopeptidase 2 | [Sites in UTR](http://www.targetscan.org/cgi-bin/targetscan/vert_60/view_gene.cgi?taxid=9606&rs=NM_006838&members=miR-1246&showcnc=1&shownc=1&showncf=1) |
| [NACC2](http://www.ncbi.nlm.nih.gov/sites/entrez?Db=gene&Cmd=ShowDetailView&TermToSearch=138151) | [NM_144653](http://www.ncbi.nlm.nih.gov/entrez/query.fcgi?cmd=Search&db=nuccore&term==NM_144653) | NACC family member 2, BEN and BTB (POZ) domain containing | [Sites in UTR](http://www.targetscan.org/cgi-bin/targetscan/vert_60/view_gene.cgi?taxid=9606&rs=NM_144653&members=miR-1246&showcnc=1&shownc=1&showncf=1) |
| [PAPOLG](http://www.ncbi.nlm.nih.gov/sites/entrez?Db=gene&Cmd=ShowDetailView&TermToSearch=64895) | [NM_022894](http://www.ncbi.nlm.nih.gov/entrez/query.fcgi?cmd=Search&db=nuccore&term==NM_022894) | poly(A) polymerase gamma | [Sites in UTR](http://www.targetscan.org/cgi-bin/targetscan/vert_60/view_gene.cgi?taxid=9606&rs=NM_022894&members=miR-1246&showcnc=1&shownc=1&showncf=1) |
| [MLL2](http://www.ncbi.nlm.nih.gov/sites/entrez?Db=gene&Cmd=ShowDetailView&TermToSearch=8085) | [NM_003482](http://www.ncbi.nlm.nih.gov/entrez/query.fcgi?cmd=Search&db=nuccore&term==NM_003482) | myeloid/lymphoid or mixed-lineage leukemia 2 | [Sites in UTR](http://www.targetscan.org/cgi-bin/targetscan/vert_60/view_gene.cgi?taxid=9606&rs=NM_003482&members=miR-1246&showcnc=1&shownc=1&showncf=1) |
| [HAUS6](http://www.ncbi.nlm.nih.gov/sites/entrez?Db=gene&Cmd=ShowDetailView&TermToSearch=54801) | [NM_017645](http://www.ncbi.nlm.nih.gov/entrez/query.fcgi?cmd=Search&db=nuccore&term==NM_017645) | HAUS augmin-like complex, subunit 6 | [Sites in UTR](http://www.targetscan.org/cgi-bin/targetscan/vert_60/view_gene.cgi?taxid=9606&rs=NM_017645&members=miR-1246&showcnc=1&shownc=1&showncf=1) |
| [SERAC1](http://www.ncbi.nlm.nih.gov/sites/entrez?Db=gene&Cmd=ShowDetailView&TermToSearch=84947) | [NM_032861](http://www.ncbi.nlm.nih.gov/entrez/query.fcgi?cmd=Search&db=nuccore&term==NM_032861) | serine active site containing 1 | [Sites in UTR](http://www.targetscan.org/cgi-bin/targetscan/vert_60/view_gene.cgi?taxid=9606&rs=NM_032861&members=miR-1246&showcnc=1&shownc=1&showncf=1) |
| [KIAA0355](http://www.ncbi.nlm.nih.gov/sites/entrez?Db=gene&Cmd=ShowDetailView&TermToSearch=9710) | [NM_014686](http://www.ncbi.nlm.nih.gov/entrez/query.fcgi?cmd=Search&db=nuccore&term==NM_014686) | KIAA0355 | [Sites in UTR](http://www.targetscan.org/cgi-bin/targetscan/vert_60/view_gene.cgi?taxid=9606&rs=NM_014686&members=miR-1246&showcnc=1&shownc=1&showncf=1) |
| [41159](http://www.ncbi.nlm.nih.gov/sites/entrez?Db=gene&Cmd=ShowDetailView&TermToSearch=989) | [NM_001011553](http://www.ncbi.nlm.nih.gov/entrez/query.fcgi?cmd=Search&db=nuccore&term==NM_001011553) | septin 7 | [Sites in UTR](http://www.targetscan.org/cgi-bin/targetscan/vert_60/view_gene.cgi?taxid=9606&rs=NM_001011553&members=miR-1246&showcnc=1&shownc=1&showncf=1) |
| [ADRB1](http://www.ncbi.nlm.nih.gov/sites/entrez?Db=gene&Cmd=ShowDetailView&TermToSearch=153) | [NM_000684](http://www.ncbi.nlm.nih.gov/entrez/query.fcgi?cmd=Search&db=nuccore&term==NM_000684) | adrenergic, beta-1-, receptor | [Sites in UTR](http://www.targetscan.org/cgi-bin/targetscan/vert_60/view_gene.cgi?taxid=9606&rs=NM_000684&members=miR-1246&showcnc=1&shownc=1&showncf=1) |
| [DLG3](http://www.ncbi.nlm.nih.gov/sites/entrez?Db=gene&Cmd=ShowDetailView&TermToSearch=1741) | [NM_001166278](http://www.ncbi.nlm.nih.gov/entrez/query.fcgi?cmd=Search&db=nuccore&term==NM_001166278) | discs, large homolog 3 (Drosophila) | [Sites in UTR](http://www.targetscan.org/cgi-bin/targetscan/vert_60/view_gene.cgi?taxid=9606&rs=NM_001166278&members=miR-1246&showcnc=1&shownc=1&showncf=1) |
| [HIPK1](http://www.ncbi.nlm.nih.gov/sites/entrez?Db=gene&Cmd=ShowDetailView&TermToSearch=204851) | [NM_181358](http://www.ncbi.nlm.nih.gov/entrez/query.fcgi?cmd=Search&db=nuccore&term==NM_181358) | homeodomain interacting protein kinase 1 | [Sites in UTR](http://www.targetscan.org/cgi-bin/targetscan/vert_60/view_gene.cgi?taxid=9606&rs=NM_181358&members=miR-1246&showcnc=1&shownc=1&showncf=1) |
| [CLASP1](http://www.ncbi.nlm.nih.gov/sites/entrez?Db=gene&Cmd=ShowDetailView&TermToSearch=23332) | [NM_001142273](http://www.ncbi.nlm.nih.gov/entrez/query.fcgi?cmd=Search&db=nuccore&term==NM_001142273) | cytoplasmic linker associated protein 1 | [Sites in UTR](http://www.targetscan.org/cgi-bin/targetscan/vert_60/view_gene.cgi?taxid=9606&rs=NM_001142273&members=miR-1246&showcnc=1&shownc=1&showncf=1) |
| [ARHGAP24](http://www.ncbi.nlm.nih.gov/sites/entrez?Db=gene&Cmd=ShowDetailView&TermToSearch=83478) | [NM_001025616](http://www.ncbi.nlm.nih.gov/entrez/query.fcgi?cmd=Search&db=nuccore&term==NM_001025616) | Rho GTPase activating protein 24 | [Sites in UTR](http://www.targetscan.org/cgi-bin/targetscan/vert_60/view_gene.cgi?taxid=9606&rs=NM_001025616&members=miR-1246&showcnc=1&shownc=1&showncf=1) |
| [AKAP6](http://www.ncbi.nlm.nih.gov/sites/entrez?Db=gene&Cmd=ShowDetailView&TermToSearch=9472) | [NM_004274](http://www.ncbi.nlm.nih.gov/entrez/query.fcgi?cmd=Search&db=nuccore&term==NM_004274) | A kinase (PRKA) anchor protein 6 | [Sites in UTR](http://www.targetscan.org/cgi-bin/targetscan/vert_60/view_gene.cgi?taxid=9606&rs=NM_004274&members=miR-1246&showcnc=1&shownc=1&showncf=1) |
| [PPP1R9B](http://www.ncbi.nlm.nih.gov/sites/entrez?Db=gene&Cmd=ShowDetailView&TermToSearch=84687) | [NM_032595](http://www.ncbi.nlm.nih.gov/entrez/query.fcgi?cmd=Search&db=nuccore&term==NM_032595) | protein phosphatase 1, regulatory (inhibitor) subunit 9B | [Sites in UTR](http://www.targetscan.org/cgi-bin/targetscan/vert_60/view_gene.cgi?taxid=9606&rs=NM_032595&members=miR-1246&showcnc=1&shownc=1&showncf=1) |
| [NRK](http://www.ncbi.nlm.nih.gov/sites/entrez?Db=gene&Cmd=ShowDetailView&TermToSearch=203447) | [NM_198465](http://www.ncbi.nlm.nih.gov/entrez/query.fcgi?cmd=Search&db=nuccore&term==NM_198465) | Nik related kinase | [Sites in UTR](http://www.targetscan.org/cgi-bin/targetscan/vert_60/view_gene.cgi?taxid=9606&rs=NM_198465&members=miR-1246&showcnc=1&shownc=1&showncf=1) |
| [GFRA2](http://www.ncbi.nlm.nih.gov/sites/entrez?Db=gene&Cmd=ShowDetailView&TermToSearch=2675) | [NM_001165038](http://www.ncbi.nlm.nih.gov/entrez/query.fcgi?cmd=Search&db=nuccore&term==NM_001165038) | GDNF family receptor alpha 2 | [Sites in UTR](http://www.targetscan.org/cgi-bin/targetscan/vert_60/view_gene.cgi?taxid=9606&rs=NM_001165038&members=miR-1246&showcnc=1&shownc=1&showncf=1) |
| [RAB31](http://www.ncbi.nlm.nih.gov/sites/entrez?Db=gene&Cmd=ShowDetailView&TermToSearch=11031) | [NM_006868](http://www.ncbi.nlm.nih.gov/entrez/query.fcgi?cmd=Search&db=nuccore&term==NM_006868) | RAB31, member RAS oncogene family | [Sites in UTR](http://www.targetscan.org/cgi-bin/targetscan/vert_60/view_gene.cgi?taxid=9606&rs=NM_006868&members=miR-1246&showcnc=1&shownc=1&showncf=1) |
| [GABRB3](http://www.ncbi.nlm.nih.gov/sites/entrez?Db=gene&Cmd=ShowDetailView&TermToSearch=2562) | [NM_000814](http://www.ncbi.nlm.nih.gov/entrez/query.fcgi?cmd=Search&db=nuccore&term==NM_000814) | gamma-aminobutyric acid (GABA) A receptor, beta 3 | [Sites in UTR](http://www.targetscan.org/cgi-bin/targetscan/vert_60/view_gene.cgi?taxid=9606&rs=NM_000814&members=miR-1246&showcnc=1&shownc=1&showncf=1) |
| [HCAR3](http://www.ncbi.nlm.nih.gov/sites/entrez?Db=gene&Cmd=ShowDetailView&TermToSearch=8843) | [NM_006018](http://www.ncbi.nlm.nih.gov/entrez/query.fcgi?cmd=Search&db=nuccore&term==NM_006018) | hydroxycarboxylic acid receptor 3 | [Sites in UTR](http://www.targetscan.org/cgi-bin/targetscan/vert_60/view_gene.cgi?taxid=9606&rs=NM_006018&members=miR-1246&showcnc=1&shownc=1&showncf=1) |
| [HCAR2](http://www.ncbi.nlm.nih.gov/sites/entrez?Db=gene&Cmd=ShowDetailView&TermToSearch=338442) | [NM_177551](http://www.ncbi.nlm.nih.gov/entrez/query.fcgi?cmd=Search&db=nuccore&term==NM_177551) | hydroxycarboxylic acid receptor 2 | [Sites in UTR](http://www.targetscan.org/cgi-bin/targetscan/vert_60/view_gene.cgi?taxid=9606&rs=NM_177551&members=miR-1246&showcnc=1&shownc=1&showncf=1) |
| [NEO1](http://www.ncbi.nlm.nih.gov/sites/entrez?Db=gene&Cmd=ShowDetailView&TermToSearch=4756) | [NM_001172623](http://www.ncbi.nlm.nih.gov/entrez/query.fcgi?cmd=Search&db=nuccore&term==NM_001172623) | neogenin 1 | [Sites in UTR](http://www.targetscan.org/cgi-bin/targetscan/vert_60/view_gene.cgi?taxid=9606&rs=NM_001172623&members=miR-1246&showcnc=1&shownc=1&showncf=1) |
| [ZMYM2](http://www.ncbi.nlm.nih.gov/sites/entrez?Db=gene&Cmd=ShowDetailView&TermToSearch=7750) | [NM_001190964](http://www.ncbi.nlm.nih.gov/entrez/query.fcgi?cmd=Search&db=nuccore&term==NM_001190964) | zinc finger, MYM-type 2 | [Sites in UTR](http://www.targetscan.org/cgi-bin/targetscan/vert_60/view_gene.cgi?taxid=9606&rs=NM_001190964&members=miR-1246&showcnc=1&shownc=1&showncf=1) |
| [MAP3K2](http://www.ncbi.nlm.nih.gov/sites/entrez?Db=gene&Cmd=ShowDetailView&TermToSearch=10746) | [NM_006609](http://www.ncbi.nlm.nih.gov/entrez/query.fcgi?cmd=Search&db=nuccore&term==NM_006609) | mitogen-activated protein kinase kinasekinase 2 | [Sites in UTR](http://www.targetscan.org/cgi-bin/targetscan/vert_60/view_gene.cgi?taxid=9606&rs=NM_006609&members=miR-1246&showcnc=1&shownc=1&showncf=1) |
| [SLITRK5](http://www.ncbi.nlm.nih.gov/sites/entrez?Db=gene&Cmd=ShowDetailView&TermToSearch=26050) | [NM_015567](http://www.ncbi.nlm.nih.gov/entrez/query.fcgi?cmd=Search&db=nuccore&term==NM_015567) | SLIT and NTRK-like family, member 5 | [Sites in UTR](http://www.targetscan.org/cgi-bin/targetscan/vert_60/view_gene.cgi?taxid=9606&rs=NM_015567&members=miR-1246&showcnc=1&shownc=1&showncf=1) |
| [LPIN2](http://www.ncbi.nlm.nih.gov/sites/entrez?Db=gene&Cmd=ShowDetailView&TermToSearch=9663) | [NM_014646](http://www.ncbi.nlm.nih.gov/entrez/query.fcgi?cmd=Search&db=nuccore&term==NM_014646) | lipin 2 | [Sites in UTR](http://www.targetscan.org/cgi-bin/targetscan/vert_60/view_gene.cgi?taxid=9606&rs=NM_014646&members=miR-1246&showcnc=1&shownc=1&showncf=1) |
| [FAM133A](http://www.ncbi.nlm.nih.gov/sites/entrez?Db=gene&Cmd=ShowDetailView&TermToSearch=286499) | [NM_001171109](http://www.ncbi.nlm.nih.gov/entrez/query.fcgi?cmd=Search&db=nuccore&term==NM_001171109) | family with sequence similarity 133, member A | [Sites in UTR](http://www.targetscan.org/cgi-bin/targetscan/vert_60/view_gene.cgi?taxid=9606&rs=NM_001171109&members=miR-1246&showcnc=1&shownc=1&showncf=1) |
| [MAFK](http://www.ncbi.nlm.nih.gov/sites/entrez?Db=gene&Cmd=ShowDetailView&TermToSearch=7975) | [NM_002360](http://www.ncbi.nlm.nih.gov/entrez/query.fcgi?cmd=Search&db=nuccore&term==NM_002360) | v-mafmusculoaponeuroticfibrosarcoma oncogene homolog K (avian) | [Sites in UTR](http://www.targetscan.org/cgi-bin/targetscan/vert_60/view_gene.cgi?taxid=9606&rs=NM_002360&members=miR-1246&showcnc=1&shownc=1&showncf=1) |
| [GPR26](http://www.ncbi.nlm.nih.gov/sites/entrez?Db=gene&Cmd=ShowDetailView&TermToSearch=2849) | [NM_153442](http://www.ncbi.nlm.nih.gov/entrez/query.fcgi?cmd=Search&db=nuccore&term==NM_153442) | G protein-coupled receptor 26 | [Sites in UTR](http://www.targetscan.org/cgi-bin/targetscan/vert_60/view_gene.cgi?taxid=9606&rs=NM_153442&members=miR-1246&showcnc=1&shownc=1&showncf=1) |
| [FAM126B](http://www.ncbi.nlm.nih.gov/sites/entrez?Db=gene&Cmd=ShowDetailView&TermToSearch=285172) | [NM_173822](http://www.ncbi.nlm.nih.gov/entrez/query.fcgi?cmd=Search&db=nuccore&term==NM_173822) | family with sequence similarity 126, member B | [Sites in UTR](http://www.targetscan.org/cgi-bin/targetscan/vert_60/view_gene.cgi?taxid=9606&rs=NM_173822&members=miR-1246&showcnc=1&shownc=1&showncf=1) |
| [TLK2](http://www.ncbi.nlm.nih.gov/sites/entrez?Db=gene&Cmd=ShowDetailView&TermToSearch=11011) | [NM_001112707](http://www.ncbi.nlm.nih.gov/entrez/query.fcgi?cmd=Search&db=nuccore&term==NM_001112707) | tousled-like kinase 2 | [Sites in UTR](http://www.targetscan.org/cgi-bin/targetscan/vert_60/view_gene.cgi?taxid=9606&rs=NM_001112707&members=miR-1246&showcnc=1&shownc=1&showncf=1) |
| [JARID2](http://www.ncbi.nlm.nih.gov/sites/entrez?Db=gene&Cmd=ShowDetailView&TermToSearch=3720) | [NM_004973](http://www.ncbi.nlm.nih.gov/entrez/query.fcgi?cmd=Search&db=nuccore&term==NM_004973) | jumonji, AT rich interactive domain 2 | [Sites in UTR](http://www.targetscan.org/cgi-bin/targetscan/vert_60/view_gene.cgi?taxid=9606&rs=NM_004973&members=miR-1246&showcnc=1&shownc=1&showncf=1) |
| [EIF2C1](http://www.ncbi.nlm.nih.gov/sites/entrez?Db=gene&Cmd=ShowDetailView&TermToSearch=26523) | [NM_012199](http://www.ncbi.nlm.nih.gov/entrez/query.fcgi?cmd=Search&db=nuccore&term==NM_012199) | eukaryotic translation initiation factor 2C, 1 | [Sites in UTR](http://www.targetscan.org/cgi-bin/targetscan/vert_60/view_gene.cgi?taxid=9606&rs=NM_012199&members=miR-1246&showcnc=1&shownc=1&showncf=1) |
| [BACH2](http://www.ncbi.nlm.nih.gov/sites/entrez?Db=gene&Cmd=ShowDetailView&TermToSearch=60468) | [NM_001170794](http://www.ncbi.nlm.nih.gov/entrez/query.fcgi?cmd=Search&db=nuccore&term==NM_001170794) | BTB and CNC homology 1, basic leucine zipper transcription factor 2 | [Sites in UTR](http://www.targetscan.org/cgi-bin/targetscan/vert_60/view_gene.cgi?taxid=9606&rs=NM_001170794&members=miR-1246&showcnc=1&shownc=1&showncf=1) |
| [FAM84A](http://www.ncbi.nlm.nih.gov/sites/entrez?Db=gene&Cmd=ShowDetailView&TermToSearch=151354) | [NM_145175](http://www.ncbi.nlm.nih.gov/entrez/query.fcgi?cmd=Search&db=nuccore&term==NM_145175) | family with sequence similarity 84, member A | [Sites in UTR](http://www.targetscan.org/cgi-bin/targetscan/vert_60/view_gene.cgi?taxid=9606&rs=NM_145175&members=miR-1246&showcnc=1&shownc=1&showncf=1) |
| [HNRNPA3](http://www.ncbi.nlm.nih.gov/sites/entrez?Db=gene&Cmd=ShowDetailView&TermToSearch=220988) | [NM_194247](http://www.ncbi.nlm.nih.gov/entrez/query.fcgi?cmd=Search&db=nuccore&term==NM_194247) | heterogeneous nuclear ribonucleoprotein A3 | [Sites in UTR](http://www.targetscan.org/cgi-bin/targetscan/vert_60/view_gene.cgi?taxid=9606&rs=NM_194247&members=miR-1246&showcnc=1&shownc=1&showncf=1) |
| [USP54](http://www.ncbi.nlm.nih.gov/sites/entrez?Db=gene&Cmd=ShowDetailView&TermToSearch=159195) | [NM_152586](http://www.ncbi.nlm.nih.gov/entrez/query.fcgi?cmd=Search&db=nuccore&term==NM_152586) | ubiquitin specific peptidase 54 | [Sites in UTR](http://www.targetscan.org/cgi-bin/targetscan/vert_60/view_gene.cgi?taxid=9606&rs=NM_152586&members=miR-1246&showcnc=1&shownc=1&showncf=1) |
| [CAV1](http://www.ncbi.nlm.nih.gov/sites/entrez?Db=gene&Cmd=ShowDetailView&TermToSearch=857) | [NM_001172895](http://www.ncbi.nlm.nih.gov/entrez/query.fcgi?cmd=Search&db=nuccore&term==NM_001172895) | caveolin 1, caveolae protein, 22kDa | [Sites in UTR](http://www.targetscan.org/cgi-bin/targetscan/vert_60/view_gene.cgi?taxid=9606&rs=NM_001172895&members=miR-1246&showcnc=1&shownc=1&showncf=1) |
| [RGS7BP](http://www.ncbi.nlm.nih.gov/sites/entrez?Db=gene&Cmd=ShowDetailView&TermToSearch=401190) | [NM_001029875](http://www.ncbi.nlm.nih.gov/entrez/query.fcgi?cmd=Search&db=nuccore&term==NM_001029875) | regulator of G-protein signaling 7 binding protein | [Sites in UTR](http://www.targetscan.org/cgi-bin/targetscan/vert_60/view_gene.cgi?taxid=9606&rs=NM_001029875&members=miR-1246&showcnc=1&shownc=1&showncf=1) |
| [CNOT2](http://www.ncbi.nlm.nih.gov/sites/entrez?Db=gene&Cmd=ShowDetailView&TermToSearch=4848) | [NM_001199302](http://www.ncbi.nlm.nih.gov/entrez/query.fcgi?cmd=Search&db=nuccore&term==NM_001199302) | CCR4-NOT transcription complex, subunit 2 | [Sites in UTR](http://www.targetscan.org/cgi-bin/targetscan/vert_60/view_gene.cgi?taxid=9606&rs=NM_001199302&members=miR-1246&showcnc=1&shownc=1&showncf=1) |
| [CTNND2](http://www.ncbi.nlm.nih.gov/sites/entrez?Db=gene&Cmd=ShowDetailView&TermToSearch=1501) | [NM_001332](http://www.ncbi.nlm.nih.gov/entrez/query.fcgi?cmd=Search&db=nuccore&term==NM_001332) | catenin (cadherin-associated protein), delta 2 (neural plakophilin-related arm-repeat protein) | [Sites in UTR](http://www.targetscan.org/cgi-bin/targetscan/vert_60/view_gene.cgi?taxid=9606&rs=NM_001332&members=miR-1246&showcnc=1&shownc=1&showncf=1) |
| [THRB](http://www.ncbi.nlm.nih.gov/sites/entrez?Db=gene&Cmd=ShowDetailView&TermToSearch=7068) | [NM_000461](http://www.ncbi.nlm.nih.gov/entrez/query.fcgi?cmd=Search&db=nuccore&term==NM_000461) | thyroid hormone receptor, beta (erythroblastic leukemia viral (v-erb-a) oncogene homolog 2, avian) | [Sites in UTR](http://www.targetscan.org/cgi-bin/targetscan/vert_60/view_gene.cgi?taxid=9606&rs=NM_000461&members=miR-1246&showcnc=1&shownc=1&showncf=1) |
| [WWTR1](http://www.ncbi.nlm.nih.gov/sites/entrez?Db=gene&Cmd=ShowDetailView&TermToSearch=25937) | [NM_001168278](http://www.ncbi.nlm.nih.gov/entrez/query.fcgi?cmd=Search&db=nuccore&term==NM_001168278) | WW domain containing transcription regulator 1 | [Sites in UTR](http://www.targetscan.org/cgi-bin/targetscan/vert_60/view_gene.cgi?taxid=9606&rs=NM_001168278&members=miR-1246&showcnc=1&shownc=1&showncf=1) |
| [HTR2C](http://www.ncbi.nlm.nih.gov/sites/entrez?Db=gene&Cmd=ShowDetailView&TermToSearch=3358) | [NM_000868](http://www.ncbi.nlm.nih.gov/entrez/query.fcgi?cmd=Search&db=nuccore&term==NM_000868) | 5-hydroxytryptamine (serotonin) receptor 2C | [Sites in UTR](http://www.targetscan.org/cgi-bin/targetscan/vert_60/view_gene.cgi?taxid=9606&rs=NM_000868&members=miR-1246&showcnc=1&shownc=1&showncf=1) |
| [MBNL3](http://www.ncbi.nlm.nih.gov/sites/entrez?Db=gene&Cmd=ShowDetailView&TermToSearch=55796) | [NM_001170701](http://www.ncbi.nlm.nih.gov/entrez/query.fcgi?cmd=Search&db=nuccore&term==NM_001170701) | muscleblind-like 3 (Drosophila) | [Sites in UTR](http://www.targetscan.org/cgi-bin/targetscan/vert_60/view_gene.cgi?taxid=9606&rs=NM_001170701&members=miR-1246&showcnc=1&shownc=1&showncf=1) |
| [RORA](http://www.ncbi.nlm.nih.gov/sites/entrez?Db=gene&Cmd=ShowDetailView&TermToSearch=6095) | [NM_002943](http://www.ncbi.nlm.nih.gov/entrez/query.fcgi?cmd=Search&db=nuccore&term==NM_002943) | RAR-related orphan receptor A | [Sites in UTR](http://www.targetscan.org/cgi-bin/targetscan/vert_60/view_gene.cgi?taxid=9606&rs=NM_002943&members=miR-1246&showcnc=1&shownc=1&showncf=1) |
| [CREB5](http://www.ncbi.nlm.nih.gov/sites/entrez?Db=gene&Cmd=ShowDetailView&TermToSearch=9586) | [NM_001011666](http://www.ncbi.nlm.nih.gov/entrez/query.fcgi?cmd=Search&db=nuccore&term==NM_001011666) | cAMP responsive element binding protein 5 | [Sites in UTR](http://www.targetscan.org/cgi-bin/targetscan/vert_60/view_gene.cgi?taxid=9606&rs=NM_001011666&members=miR-1246&showcnc=1&shownc=1&showncf=1) |
| [TSHZ2](http://www.ncbi.nlm.nih.gov/sites/entrez?Db=gene&Cmd=ShowDetailView&TermToSearch=128553) | [NM_001193421](http://www.ncbi.nlm.nih.gov/entrez/query.fcgi?cmd=Search&db=nuccore&term==NM_001193421) | teashirt zinc finger homeobox 2 | [Sites in UTR](http://www.targetscan.org/cgi-bin/targetscan/vert_60/view_gene.cgi?taxid=9606&rs=NM_001193421&members=miR-1246&showcnc=1&shownc=1&showncf=1) |
| [BCL9](http://www.ncbi.nlm.nih.gov/sites/entrez?Db=gene&Cmd=ShowDetailView&TermToSearch=607) | [NM_004326](http://www.ncbi.nlm.nih.gov/entrez/query.fcgi?cmd=Search&db=nuccore&term==NM_004326) | B-cell CLL/lymphoma 9 | [Sites in UTR](http://www.targetscan.org/cgi-bin/targetscan/vert_60/view_gene.cgi?taxid=9606&rs=NM_004326&members=miR-1246&showcnc=1&shownc=1&showncf=1) |
| [VPS53](http://www.ncbi.nlm.nih.gov/sites/entrez?Db=gene&Cmd=ShowDetailView&TermToSearch=55275) | [NM_001128159](http://www.ncbi.nlm.nih.gov/entrez/query.fcgi?cmd=Search&db=nuccore&term==NM_001128159) | vacuolar protein sorting 53 homolog (S. cerevisiae) | [Sites in UTR](http://www.targetscan.org/cgi-bin/targetscan/vert_60/view_gene.cgi?taxid=9606&rs=NM_001128159&members=miR-1246&showcnc=1&shownc=1&showncf=1) |
| [CACNG2](http://www.ncbi.nlm.nih.gov/sites/entrez?Db=gene&Cmd=ShowDetailView&TermToSearch=10369) | [NM_006078](http://www.ncbi.nlm.nih.gov/entrez/query.fcgi?cmd=Search&db=nuccore&term==NM_006078) | calcium channel, voltage-dependent, gamma subunit 2 | [Sites in UTR](http://www.targetscan.org/cgi-bin/targetscan/vert_60/view_gene.cgi?taxid=9606&rs=NM_006078&members=miR-1246&showcnc=1&shownc=1&showncf=1) |
| [CELF2](http://www.ncbi.nlm.nih.gov/sites/entrez?Db=gene&Cmd=ShowDetailView&TermToSearch=10659) | [NM_001025076](http://www.ncbi.nlm.nih.gov/entrez/query.fcgi?cmd=Search&db=nuccore&term==NM_001025076) | CUGBP, Elav-like family member 2 | [Sites in UTR](http://www.targetscan.org/cgi-bin/targetscan/vert_60/view_gene.cgi?taxid=9606&rs=NM_001025076&members=miR-1246&showcnc=1&shownc=1&showncf=1) |
| [C10orf26](http://www.ncbi.nlm.nih.gov/sites/entrez?Db=gene&Cmd=ShowDetailView&TermToSearch=54838) | [NM_001083913](http://www.ncbi.nlm.nih.gov/entrez/query.fcgi?cmd=Search&db=nuccore&term==NM_001083913) | chromosome 10 open reading frame 26 | [Sites in UTR](http://www.targetscan.org/cgi-bin/targetscan/vert_60/view_gene.cgi?taxid=9606&rs=NM_001083913&members=miR-1246&showcnc=1&shownc=1&showncf=1) |
| [KLHDC10](http://www.ncbi.nlm.nih.gov/sites/entrez?Db=gene&Cmd=ShowDetailView&TermToSearch=23008) | [NM_014997](http://www.ncbi.nlm.nih.gov/entrez/query.fcgi?cmd=Search&db=nuccore&term==NM_014997) | kelch domain containing 10 | [Sites in UTR](http://www.targetscan.org/cgi-bin/targetscan/vert_60/view_gene.cgi?taxid=9606&rs=NM_014997&members=miR-1246&showcnc=1&shownc=1&showncf=1) |
| [ZBTB40](http://www.ncbi.nlm.nih.gov/sites/entrez?Db=gene&Cmd=ShowDetailView&TermToSearch=9923) | [NM_001083621](http://www.ncbi.nlm.nih.gov/entrez/query.fcgi?cmd=Search&db=nuccore&term==NM_001083621) | zinc finger and BTB domain containing 40 | [Sites in UTR](http://www.targetscan.org/cgi-bin/targetscan/vert_60/view_gene.cgi?taxid=9606&rs=NM_001083621&members=miR-1246&showcnc=1&shownc=1&showncf=1) |
| [TMEM182](http://www.ncbi.nlm.nih.gov/sites/entrez?Db=gene&Cmd=ShowDetailView&TermToSearch=130827) | [NM_144632](http://www.ncbi.nlm.nih.gov/entrez/query.fcgi?cmd=Search&db=nuccore&term==NM_144632) | transmembrane protein 182 | [Sites in UTR](http://www.targetscan.org/cgi-bin/targetscan/vert_60/view_gene.cgi?taxid=9606&rs=NM_144632&members=miR-1246&showcnc=1&shownc=1&showncf=1) |
| [CPLX2](http://www.ncbi.nlm.nih.gov/sites/entrez?Db=gene&Cmd=ShowDetailView&TermToSearch=10814) | [NM_001008220](http://www.ncbi.nlm.nih.gov/entrez/query.fcgi?cmd=Search&db=nuccore&term==NM_001008220) | complexin 2 | [Sites in UTR](http://www.targetscan.org/cgi-bin/targetscan/vert_60/view_gene.cgi?taxid=9606&rs=NM_001008220&members=miR-1246&showcnc=1&shownc=1&showncf=1) |
| [DPY19L3](http://www.ncbi.nlm.nih.gov/sites/entrez?Db=gene&Cmd=ShowDetailView&TermToSearch=147991) | [NM_001172774](http://www.ncbi.nlm.nih.gov/entrez/query.fcgi?cmd=Search&db=nuccore&term==NM_001172774) | dpy-19-like 3 (C. elegans) | [Sites in UTR](http://www.targetscan.org/cgi-bin/targetscan/vert_60/view_gene.cgi?taxid=9606&rs=NM_001172774&members=miR-1246&showcnc=1&shownc=1&showncf=1) |
| [SLC24A2](http://www.ncbi.nlm.nih.gov/sites/entrez?Db=gene&Cmd=ShowDetailView&TermToSearch=25769) | [NM_001193288](http://www.ncbi.nlm.nih.gov/entrez/query.fcgi?cmd=Search&db=nuccore&term==NM_001193288) | solute carrier family 24 (sodium/potassium/calcium exchanger), member 2 | [Sites in UTR](http://www.targetscan.org/cgi-bin/targetscan/vert_60/view_gene.cgi?taxid=9606&rs=NM_001193288&members=miR-1246&showcnc=1&shownc=1&showncf=1) |
| [NFIB](http://www.ncbi.nlm.nih.gov/sites/entrez?Db=gene&Cmd=ShowDetailView&TermToSearch=4781) | [NM_001190737](http://www.ncbi.nlm.nih.gov/entrez/query.fcgi?cmd=Search&db=nuccore&term==NM_001190737) | nuclear factor I/B | [Sites in UTR](http://www.targetscan.org/cgi-bin/targetscan/vert_60/view_gene.cgi?taxid=9606&rs=NM_001190737&members=miR-1246&showcnc=1&shownc=1&showncf=1) |
| [SPOCK2](http://www.ncbi.nlm.nih.gov/sites/entrez?Db=gene&Cmd=ShowDetailView&TermToSearch=9806) | [NM_001134434](http://www.ncbi.nlm.nih.gov/entrez/query.fcgi?cmd=Search&db=nuccore&term==NM_001134434) | sparc/osteonectin, cwcv and kazal-like domains proteoglycan (testican) 2 | [Sites in UTR](http://www.targetscan.org/cgi-bin/targetscan/vert_60/view_gene.cgi?taxid=9606&rs=NM_001134434&members=miR-1246&showcnc=1&shownc=1&showncf=1) |
| [NFIA](http://www.ncbi.nlm.nih.gov/sites/entrez?Db=gene&Cmd=ShowDetailView&TermToSearch=4774) | [NM_001134673](http://www.ncbi.nlm.nih.gov/entrez/query.fcgi?cmd=Search&db=nuccore&term==NM_001134673) | nuclear factor I/A | [Sites in UTR](http://www.targetscan.org/cgi-bin/targetscan/vert_60/view_gene.cgi?taxid=9606&rs=NM_001134673&members=miR-1246&showcnc=1&shownc=1&showncf=1) |
| [PLAGL2](http://www.ncbi.nlm.nih.gov/sites/entrez?Db=gene&Cmd=ShowDetailView&TermToSearch=5326) | [NM_002657](http://www.ncbi.nlm.nih.gov/entrez/query.fcgi?cmd=Search&db=nuccore&term==NM_002657) | pleiomorphic adenoma gene-like 2 | [Sites in UTR](http://www.targetscan.org/cgi-bin/targetscan/vert_60/view_gene.cgi?taxid=9606&rs=NM_002657&members=miR-1246&showcnc=1&shownc=1&showncf=1) |
| [ANKH](http://www.ncbi.nlm.nih.gov/sites/entrez?Db=gene&Cmd=ShowDetailView&TermToSearch=56172) | [NM_054027](http://www.ncbi.nlm.nih.gov/entrez/query.fcgi?cmd=Search&db=nuccore&term==NM_054027) | ankylosis, progressive homolog (mouse) | [Sites in UTR](http://www.targetscan.org/cgi-bin/targetscan/vert_60/view_gene.cgi?taxid=9606&rs=NM_054027&members=miR-1246&showcnc=1&shownc=1&showncf=1) |
| [PURB](http://www.ncbi.nlm.nih.gov/sites/entrez?Db=gene&Cmd=ShowDetailView&TermToSearch=5814) | [NM_033224](http://www.ncbi.nlm.nih.gov/entrez/query.fcgi?cmd=Search&db=nuccore&term==NM_033224) | purine-rich element binding protein B | [Sites in UTR](http://www.targetscan.org/cgi-bin/targetscan/vert_60/view_gene.cgi?taxid=9606&rs=NM_033224&members=miR-1246&showcnc=1&shownc=1&showncf=1) |
| [CHTF8](http://www.ncbi.nlm.nih.gov/sites/entrez?Db=gene&Cmd=ShowDetailView&TermToSearch=54921) | [NM_001039690](http://www.ncbi.nlm.nih.gov/entrez/query.fcgi?cmd=Search&db=nuccore&term==NM_001039690) | CTF8, chromosome transmission fidelity factor 8 homolog (S. cerevisiae) | [Sites in UTR](http://www.targetscan.org/cgi-bin/targetscan/vert_60/view_gene.cgi?taxid=9606&rs=NM_001039690&members=miR-1246&showcnc=1&shownc=1&showncf=1) |
| [C9orf47](http://www.ncbi.nlm.nih.gov/sites/entrez?Db=gene&Cmd=ShowDetailView&TermToSearch=286223) | [NM_001001938](http://www.ncbi.nlm.nih.gov/entrez/query.fcgi?cmd=Search&db=nuccore&term==NM_001001938) | chromosome 9 open reading frame 47 | [Sites in UTR](http://www.targetscan.org/cgi-bin/targetscan/vert_60/view_gene.cgi?taxid=9606&rs=NM_001001938&members=miR-1246&showcnc=1&shownc=1&showncf=1) |
| [PRKCE](http://www.ncbi.nlm.nih.gov/sites/entrez?Db=gene&Cmd=ShowDetailView&TermToSearch=5581) | [NM_005400](http://www.ncbi.nlm.nih.gov/entrez/query.fcgi?cmd=Search&db=nuccore&term==NM_005400) | protein kinase C, epsilon | [Sites in UTR](http://www.targetscan.org/cgi-bin/targetscan/vert_60/view_gene.cgi?taxid=9606&rs=NM_005400&members=miR-1246&showcnc=1&shownc=1&showncf=1) |
| [CTC1](http://www.ncbi.nlm.nih.gov/sites/entrez?Db=gene&Cmd=ShowDetailView&TermToSearch=80169) | [NM_025099](http://www.ncbi.nlm.nih.gov/entrez/query.fcgi?cmd=Search&db=nuccore&term==NM_025099) | CTS telomere maintenance complex component 1 | [Sites in UTR](http://www.targetscan.org/cgi-bin/targetscan/vert_60/view_gene.cgi?taxid=9606&rs=NM_025099&members=miR-1246&showcnc=1&shownc=1&showncf=1) |
| [PDPK1](http://www.ncbi.nlm.nih.gov/sites/entrez?Db=gene&Cmd=ShowDetailView&TermToSearch=5170) | [NM_002613](http://www.ncbi.nlm.nih.gov/entrez/query.fcgi?cmd=Search&db=nuccore&term==NM_002613) | 3-phosphoinositide dependent protein kinase-1 | [Sites in UTR](http://www.targetscan.org/cgi-bin/targetscan/vert_60/view_gene.cgi?taxid=9606&rs=NM_002613&members=miR-1246&showcnc=1&shownc=1&showncf=1) |
| [EBF1](http://www.ncbi.nlm.nih.gov/sites/entrez?Db=gene&Cmd=ShowDetailView&TermToSearch=1879) | [NM_024007](http://www.ncbi.nlm.nih.gov/entrez/query.fcgi?cmd=Search&db=nuccore&term==NM_024007) | early B-cell factor 1 | [Sites in UTR](http://www.targetscan.org/cgi-bin/targetscan/vert_60/view_gene.cgi?taxid=9606&rs=NM_024007&members=miR-1246&showcnc=1&shownc=1&showncf=1) |
| [DGCR2](http://www.ncbi.nlm.nih.gov/sites/entrez?Db=gene&Cmd=ShowDetailView&TermToSearch=9993) | [NM_001173533](http://www.ncbi.nlm.nih.gov/entrez/query.fcgi?cmd=Search&db=nuccore&term==NM_001173533) | DiGeorge syndrome critical region gene 2 | [Sites in UTR](http://www.targetscan.org/cgi-bin/targetscan/vert_60/view_gene.cgi?taxid=9606&rs=NM_001173533&members=miR-1246&showcnc=1&shownc=1&showncf=1) |
| [ZADH2](http://www.ncbi.nlm.nih.gov/sites/entrez?Db=gene&Cmd=ShowDetailView&TermToSearch=284273) | [NM_175907](http://www.ncbi.nlm.nih.gov/entrez/query.fcgi?cmd=Search&db=nuccore&term==NM_175907) | zinc binding alcohol dehydrogenase domain containing 2 | |
