# Supplementary material for: Parallel mRNA and MicroRNA Profiling of HEV71-Infected Human Neuroblastoma Cells Reveal the Up-Regulation of miR-1246 in Association with DLG3 Repression
Source: PLoS One. 2014 Apr 16;9(4):e95272. doi: 10.1371/journal.pone.0095272 (PMC3989279; doi:10.1371/journal.pone.0095272)
Supplement: Table S5 — Co-occurrence genes and detection primers used for qRT-PCR analysis. (DOCX) [file pone.0095272.s006.docx]

**Table S5. Co-occurrence genes and detection primers used for qRT-PCR analysis**

| Co-occurrence genes | Primer sequence (5’ →3’) | |
| --- | --- | --- |
|  | Sense | Antisense |
| VPS53 | GTGGATGTAAGTCTGATTGA | AATACTGCTGGAGATGAC |
| NFIB | TATCACAGTATCAGTTAAGG | CATTGAAGACTCCAGATT |
| DLG3 | GTGATAATGAGGTGGATGGA | GATGAACTTGTTGTCCTGAA |
| GRHL1 | AGTTAAGGCATTGGAAGT | CGATGTTACTGATAGTGTTG |
| KIAA0240 | ACAGACATTGGCAGAAGA | GCTGATGAAGAAGGATGAAG |
| CCNG2 | ACCATCTGTATTAGCCTTGTG | CAGTAGAAGAACTCAGTGTCAT |
| ADRB1 | AGTACGGCTCCTTCTTCT | AATGACACACAGGGTCTC |
| SLC12A2 | ATCCTCAGTCAGCCATAC | AACACAAGAACCTACAGATAC |
| CREB5 | AGAATGAAGTGTCTATGT | GGAAGTAGTGATGGTATT |
| DNAJC3 | GCCTTCCTTGATAAGATT | GTCTCCTAGTTGGTAGTA |
| MCCC2 | GAGCACTTCACATATCAA | ACCTCCTCATTGTCATAT |
| PSD3 | ATCAGTCACTCAGGTATT | ATCGGTATTAAGAAGCATTA |
| CXorf36 | CTTTCCTCGGTCTTGATAAATGC | GCCAGCCAGTTGTCAGAT |
| C6orf35 | CTGGTTAAAGGTGGAATT | GCCAATGATAATGTTGTAAT |
| GAPDH* | AGAAGGCTGGGGCTCATTTG | AGGGGCCATCCACAGTCTTC |

*Housekeeping gene transcript serving as the normalization control for real-time RT-PCR.

Other genes were selected from the microarray analysis for real-time RT-PCR
